# Acupuncture as an adjunctive therapy on embryo transfer day: a systematic review and meta-analysis of clinical pregnancy and live birth outcomes

**DOI:** 10.3389/frph.2025.1673144

**Published:** 2025-09-23

**Authors:** Yixin Wang, Jing Ji, Na Duan, Yanyun Yin

**Affiliations:** ^1^ Department of Reproductive Medicine, Affiliated Hospital of Nanjing University of Chinese Medicine, Nanjing, Jiangsu, China; ^2^ Department of Gynecology, Affiliated Hospital of Nanjing University of Chinese Medicine, Nanjing, Jiangsu, China; ^3^ Department of Radiology, Affiliated Hospital of Nanjing University of Chinese Medicine, Nanjing, Jiangsu, China

**Keywords:** assisted reproductive technology, embryo transfer, infertility, acupuncture, clinical pregnancy rate, live birth, systematic review, meta-analysis

## Abstract

**Background:**

Acupuncture is frequently employed during the process of embryo transfer. Nevertheless, its precise function in enhancing the likelihood of successful clinical pregnancy or live birth remains ambiguous.

**Objective:**

To evaluate the efficacy of acupuncture as a complementary intervention to embryo transfer in managing female subfertility, compare the effects of acupuncture vs. sham acupuncture on clinical pregnancy rate in assisted reproductive technology, and clarify the optimal timing of acupuncture administration within *in vitro* fertilization protocols.

**Methods:**

All literatures which described randomized controlled trials of acupuncture during the process of embryo transfer were obtained through searches of Cochrane Central, PubMed and Embase database (all to May 2025). Eleven randomized controlled trials were incorporated into the review. Selection of studies, quality assessment and data extraction were carried out independently by two review authors. Meta analysis was conducted, incorporating both risk ratios and 95% confidence intervals. The primary outcome measure was the clinical pregnancy rate, defined as the proportion of patients with an intrauterine gestational sac confirmed by ultrasound with or without a fetal heart. The secondary outcome measure was the live birth rate, defined as the proportion of patients with a pregnancy lasting ≥20 weeks or a birth weight of at least 400 g.

**Results:**

Eleven studies were selected for review, and nine of these were deemed acceptable based on their discussion of clinical pregnancy rate. By combining the studies and analyzing the results, it was concluded that acupuncture has demonstrated a positive impact on clinical pregnancy rate in contrast with the blank control group [1.25 (1.05–1.50), *P* = 0.013]. A statistical analysis revealed no significant differences between the sham acupuncture group and the acupuncture group [1.01(0.87–1.17), *P* = 0.907]. No statistically significant discrepancy between the Pulus Protocol [1.083(0.946–1.240)] and Delphi Consensus [1.164(0.938–1.445)]. Acupuncture has no positive impact on live birth rate during embryo transfer [1.01(0.88–1.15), *P* = 0.930]).

**Conclusions:**

The results of this meta-analysis suggest that a positive correlation has been demonstrated between acupuncture and clinical pregnancy rate during embryo transfer when compared to the blank control group; however, this advantage does not hold when compared to the use of sham acupuncture. The Delphi Consensus revealed no discrepancy in clinical pregnancy rate when compared with the Pulus Protocol. It should be noted that the impact of acupuncture on live birth rate remains to be elucidated.

**Systematic Review Registration:**

https://www.crd.york.ac.uk/PROSPERO/view/CRD420251067805, identifier (CRD420251067805).

## Introduction

The application of reproductive technologies, most notably *in vitro* fertilization (IVF), has engendered considerable optimism amongst couples struggling with infertility on a global scale. Despite significant progress in the optimization of stimulation protocols for multi-follicular development and the advent of novel technologies for embryo quality assessment, the success rate for IVF remains low on a global scale.

Embryo implantation represents a pivotal step in the process of reproductive success, yet the phenomenon of implantation failure remains an unsolved problem in the field of assisted reproductive technology (ART). The success of embryo transfer (ET) is influenced by a number of factors, including the psychological well-being of the patient and the physician's experience. There has been mounting attention on the role of endometrial thickness and endometrial receptivity with the window of implantation as an expanded area of interest (1).

Embryo cryopreservation was developed as a means of preserving surplus embryos subsequent to the initial transfer of fresh embryos. A systematic review demonstrated that the utilization of in frozen embryo transfer (FET), in comparison with fresh embryo transfer, resulted in a substantial enhancement in clinical and ongoing pregnancy rates among patients undergoing ART (2). However, although FET is generally safe, potential injury to embryos during freezing and thawing is possible (3). Conversely, FET may incur an increased cost of treatment and workload, necessitating additional embryo manipulation procedures (4).

It has been demonstrated that the success rate of fresh embryo transfer in IVF cycles is suboptimal. The most recent results obtained from European registers by ESHRE demonstrate that the clinical pregnancy rate (CPR) per fresh transfer following IVF is an average of 32%, thus indicating that the implantation of embryos and the subsequent maintenance of a pregnancy are far from guaranteed (5).

Acupuncture has been utilized as a therapeutic modality for female reproductive disease, founded on the philosophy of vital energy circulation throughout the body via specific meridians. The potential mechanisms of acupuncture include increased blood flow to the uterus and the alleviation of anxiety and stress (6).

A multi-center randomized controlled trial (RCT) (7) published in the Journal of the American Medical Association (JAMA) investigated the effect of acupuncture vs. sham acupuncture on live birth rate (LBR) in IVF. However, it is important to note that the results were not entirely satisfactory. The Delphi Consensus (8), founded on the principles of the Pulus Protocol constitutes a significant approach to clinical decision-making. It is particularly recommended for developing clinical practice guidelines.

An updated review (9) has revealed that there is an absence of evidence to suggest that acupuncture administered in proximity to the scheduled ET improves the LBR.

The integration of acupuncture into mainstream medicine is contingent upon the substantiation of its efficacy. Despite the fact that sham acupuncture is regarded as an acceptable form of control, true patient blinding has been demonstrated to present a considerable challenge. In the context of RCTs, the focus of studies in this area should be on the utilization of “standardized” acupuncture methods, thereby facilitating the establishment of valid comparisons.

In view of the controversy surrounding this topic, it is imperative to undertake this systematic review and meta-analysis in order to ascertain the efficacy of acupuncture as a complementary treatment to ET in the management of female subfertility.

## Material and methods

This systematic review and meta-analysis was conducted in accordance with the PRISMA guidelines (10).

### Information sources

A systematic computerized literature search of articles published from inception until 18 May 2025 was conducted for all published articles in the databases PubMed, Embase and Cochrane Central. The complete search string is as follows: [“acupuncture(MeSH Terms)” OR acupuncture] AND [“embryo transfer(MeSH Terms)” OR “embryo transfer”] AND “randomized controlled trial[pt]” AND “English[lang]” AND (inception[Date-Publication]: “2025/05/18”[Date-Publication]). An exhaustive search history is provided in Supplementary Appendix 1. Subsequently, the reference list of the eligible studies was screened for additional relevant articles.

### Study selection and data extraction

#### Eligibility criteria

Inclusion criteria were women aged 18–42 years undergoing a fresh IVF or intracytoplasmic sperm injection cycle and not using acupuncture. To minimize the risk of selection bias and ensure the reliability of data extraction, our literature search was restricted to English-language studies. RCTs reported in English without any regional restrictions will be included. In the event of a randomized crossover trial, the inclusion of results will be restricted to the first phase alone.

Exclusion criteria were meta-analyses, case reports, animal experimental studies, quasi-RCTs, expert experience, and studies with lack of relevance or insufficient data reporting.

Acupuncture will be defined in this review including manual acupuncture, auricular acupuncture, auricular acupressure. Conversely, interventions involving electro-acupuncture, acupoint application, moxibustion, catgut embedding, transcutaneous electrical acupoint stimulation, acupoint injection, and related modalities are to be excluded from consideration. Sterile disposable stainless steel needles were inserted to tissue level and manipulated until the requisite sensation was obtained, which could be described as a feeling of soreness or numbness, distension, or pain. Treatments in the control groups will include no treatment, sham acupuncture, pharmacotherapy, waiting list, or usual care.

#### Screening of studies

In accordance with the exclusion and inclusion criteria, references imported into Endnote 21 software will undergo screening subsequent to the exclusion of duplicated publications.

Two reviewers (WYX and JJ) independently carried out the study selection and data extraction from the eligible studies. Both reviewers received prior training on the Cochrane Risk of Bias 2.0 (RoB 2.0) tool to ensure consistent interpretation of criteria. Discrepancies between reviewers during appraisal were resolved through discussion with a third senior reviewer (DN) who was blinded to the initial evaluations. Data extracted included: author, year of publication, number of participants, acupuncture timing, points, outcomes and treatments in the control groups. The data will ultimately be transferred to both RevMan and STATA software.

#### Assessment of risk of bias

**Figure 1:**
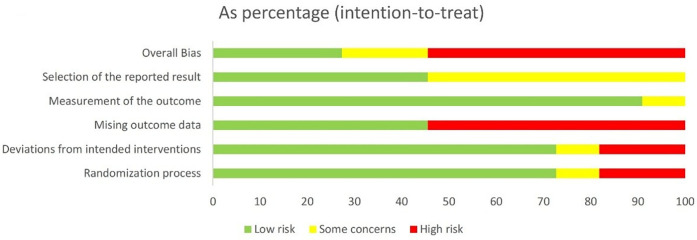
Risk of bias graph.

**Figure 2:**
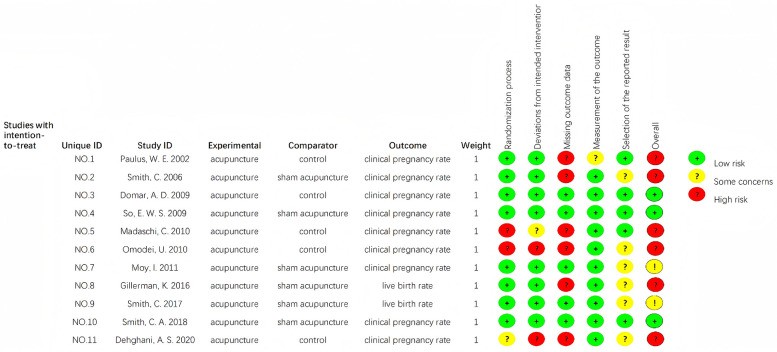
Risk of bias summary.

The methodological quality of included RCTs was appraised using the Cochrane RoB 2.0 tool (11), a gold-standard instrument for evaluating RCT methodological rigor. Appraisal focused on five core domains of bias risk: (1) random sequence generation, (2) allocation concealment, (3) blinding of participants and personnel, (4) completeness of outcome data, and (5) selective reporting, with an additional assessment of “other sources of bias”. The risk of bias is then assessed by each outcome, with the risk being categorized as low, high or some concerns. Detailed quality appraisal results for each included study—including specific judgments and justifications for ambiguous cases—are presented in Figures 1, 2.

To ensure transparency in our RoB assessment and enable readers to validate domain-level judgments, we conducted a systematic evaluation of all included studies using the Cochrane RoB 2 tool—focusing on five core domains: random sequence generation, allocation concealment, blinding of participants and personnel, completeness of outcome data, and selective reporting (Table 1).

**Table 1:** Detailed cochrane RoB 2 assessment for each included study.

Study ID	Random sequence generation	Allocation concealment	Blinding of participants and personnel	Completeness of outcome data
Paulus, W. E. 2022	Low risk	Low risk	Unclear risk	High risk
Smith, C. 2006	Low risk	Unclear risk	Unclear risk	High risk
Domar, A. D. 2009	Low risk	Low risk	Low risk	High risk
So, E. W. S. 2009	Low risk	Low risk	Low risk	Unclear risk
Madaschi, C. 2010	Low risk	Unclear risk	Unclear risk	High risk
Omodei, U. 2010	Unclear risk	Unclear risk	High risk	High risk
Moy, I. 2011	Low risk	Low risk	Low risk	High risk
Gillerman, K. 2016	Low risk	Unclear risk	High risk	Unclear risk
Smith, C. 2017	Low risk	Low risk	Low risk	High risk
Smith, C. A. 2018	Low risk	Low risk	Low risk	Unclear risk
Dehghani, A. S. 2020	Unclear risk	Unclear risk	High risk	High risk

### Statistical analyses

#### Outcomes

Studies reporting one or more of the below-mentioned outcomes will be included. Otherwise, the trial will be excluded. The primary outcome was clinical pregnancy. The definition of clinical pregnancy was amended *post hoc* to the definition used by the National Perinatal Epidemiology and Statistics Unit, defined as evidence on ultrasonography of an intrauterine sac with or without a fetal heart. The secondary outcome was a live birth, defined as the delivery of one or more living infants with a gestational age of more than 20 weeks or a birth weight of at least 400 g. For dichotomous outcomes, the risk ratios (RR) was calculated, with the 95% confidence intervals (CI) subsequently determined. Of the 11 studies included in this analysis, 6 studies did not report adverse events or withdrawals due to adverse events.

#### Assessment of heterogeneity

First, heterogeneity across included studies was evaluated using the *I*^2^ statistic and Cochran's *Q* test. Specifically, a fixed-effect model was employed for meta-analysis in cases of low to moderate heterogeneity, defined as an *I*^2^ statistic ≤50% and a *P*-value ≥0.10 for Cochran's *Q* test. In contrast, where significant heterogeneity was identified (*I*^2^ statistic > 50% or *P*-value <0.10 for Cochran's *Q* test), a random-effect model was utilized; additionally, subgroup analyses (stratified by the varying acupuncture timing and diverse treatments) and sensitivity analyses (excluding studies of low methodological quality) were conducted to explore potential sources of this heterogeneity. The findings from these heterogeneity assessments were then used to contextualize and interpret the reliability of the statistical effect sizes derived from the meta-analyses.

This study incorporated several conference abstract-only RCTs. Although sensitivity analyses indicated that these abstracts did not materially alter the core conclusions, their inherent limitations—specifically incomplete data reporting and limited methodological detail—should be acknowledged, as these may have marginally compromised the precision of the results. Future research ought to prioritize fully published studies to validate these findings.

#### Assessment of publication bias

The comparison-adjusted funnel plot is a visualization tool employed to inspect reported bias and small-scale effects. Egger's regression test will be used to analyze the causes of asymmetry.

## Results

**Figure 3:**
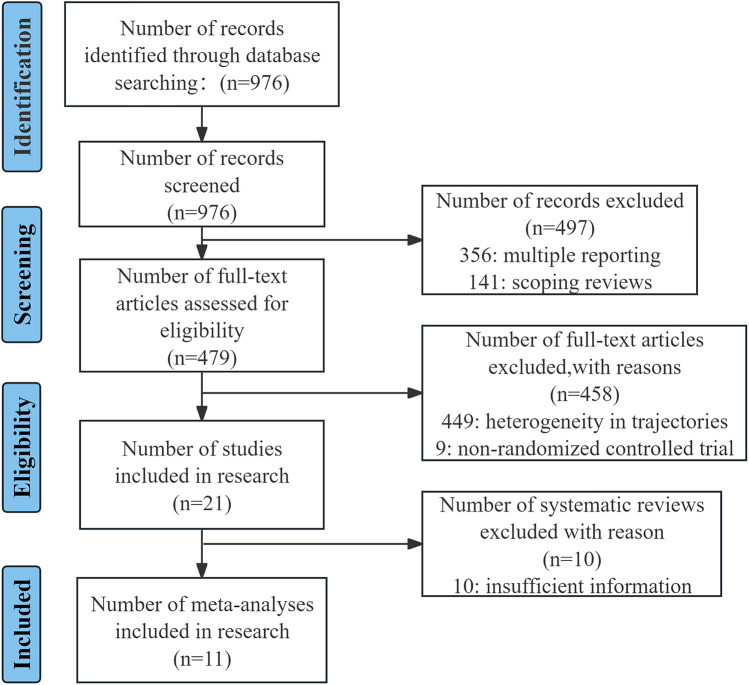
The PRISMA flow diagram of the study selection process.

As demonstrated in Figure 3, the following flowchart illustrates the studies that were identified during the literature search.

The final analysis comprised a total of 11 studies (7, 12–21), published from inception until 18 May 2025, among a total of 3,561 women. Table 2 provides a summary of the characteristics of each study that is represented in the subsequent analysis. To contextualize the heterogeneity of acupuncture protocols across included trials and enhance the reproducibility of our findings, Table 3 provides a comprehensive summary of intervention characteristics for each RCT included in this meta-analysis.

**Table 2:** Basic characteristics of the included references.

Author	Year	Sample size (A/C)	Acupuncture timing	Outcomes	Treatments in the control groups	Points
Paulus, W. E.	2002	80/80	Pulus	Clinical pregnancy	No-intervention	Cx6, Sp8, Liv3, Gv20, S29, S36, Sp6, Sp10, Li4
Smith, C.	2006	110/118	Delphi	Clinical pregnancy	Sham acupuncture	Cx6, Sp8, Liv3, S29, S36, Sp6, Sp10
Domar, A. D.	2009	78/68	Pulus	Clinical pregnancy	No-intervention	Cx6, Sp8, Liv3, Gv20, S29, S36, Sp6, Sp10, Li4
So, E. W. S.	2009	185/185	Pulus	Clinical pregnancy, live birth	Sham acupuncture	PC6, SP8, LR3, GV20, ST29, ST36, SP6, SP10, LI4
Madaschi, C.	2010	208/208	Pulus	Clinical pregnancy, live birth	No-intervention	PC6, SP8, LR3, GV20, ST29(before ET); ST36, SP6, SP10, Li4(after ET)
Omodei, U.	2010	84/84	Pulus	Clinical pregnancy	No-intervention	EX-HN3, LI4, ST36, SP6, LR3 and auricular point Shenmen and Zhigong
Moy, I.	2011	86/74	Pulus	Clinical pregnancy	Sham acupuncture	CV6, SP8, LIV3, ST29, DU20, ST36, SP6, SP10, LI4
Gillerman, K.	2016	67/60	Delphi	-	No-intervention	—
Smith, C.	2017	424/424	Delphi	Live birth	Sham acupuncture	ST29, Ren4, Ren6, SP6, SP10, SP8, SP10, LR3, Ren4, HT7, PC6, DU20, KD3, ST36, SP6 and auricular points Shenmen and Zhigong
Smith, C. A.	2018	408/406	Delphi	Clinical pregnancy, live birth	Sham acupuncture	ST29, Ren4, Ren6, SP6, SP10, ST29, SP8, LR3, Ren4, HT7, PC6 and auricular point Zhigong (before ET); DU20, KD3, ST36, SP6, PC6 and auricular point ShenMen (after ET)
Dehghani, A. S.	2020	62/62	Pulus	Clinical pregnancy	No-intervention	Ht7, PC6, Ren6, Du20, SP6, Ren4

**Table 3:** Detailed acupuncture intervention characteristics of included trials.

Study ID	Needle retention time	Depth	Practitioner qualification
Paulus, W. E. 2022	25 min	10–20 mm	The same well-trained examiner in the same way
Smith, C. 2006	25 min	Not mentioned	Two acupuncturists administered acupuncture treatments, with the majority being administered by the primary acupuncture researcher
Domar, A. D. 2009	25 min	Not mentioned	The acupuncturist who has extensive clinical experience
So, E. W. S. 2009	25 min	10–20 mm	All acupuncture treatments were performed in the same way by the same certified acupuncturist
Madaschi, C. 2010	25 min	10–20 mm	Not mentioned
Omodei, U. 2010	Not mentioned	Not mentioned	Not mentioned
Moy, I. 2011	25 min	Not mentioned	Hospital-employed acupuncturists
Gillerman, K. 2016	Not mentioned	Not mentioned	Not mentioned
Smith, C. 2017	Not mentioned	Not mentioned	Not mentioned
Smith, C. A. 2018	25 min	Not mentioned	All acupuncturists were trained in the treatment protocol with annual refresher training provided
Dehghani, A. S. 2020	25 min	Not mentioned	Not mentioned

### Commonly used meridians and acupoints

**Figure 4:**
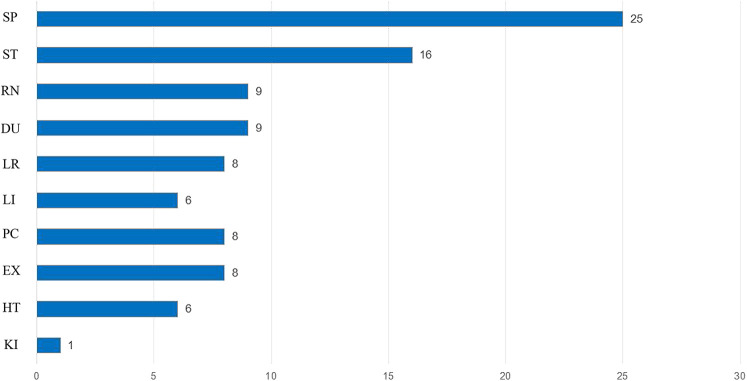
Commonly used meridians frequency (*N* = time).

**Figure 5:**
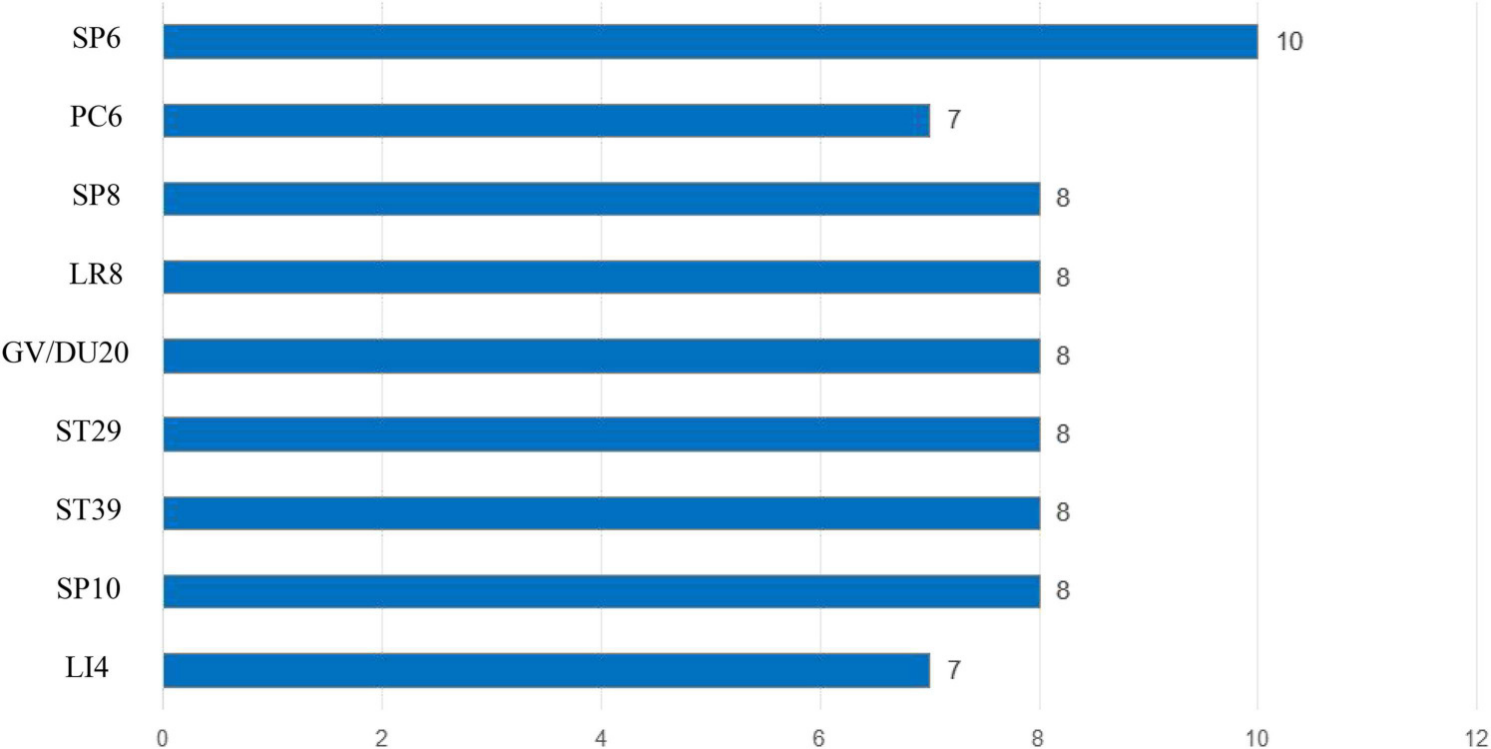
Commonly used acupoints frequency (*N* = time).

The findings of this meta-analysis indicated that the most frequently utilized meridians were SP, ST, and RN, while the three most commonly employed acupoints were SP6, PC6, and SP8 (Figures 4, 5).

### Meta-analysis

**Figure 6:**
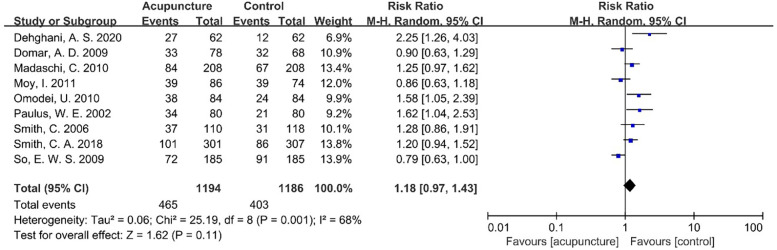
Meta-analysis forest plot for CPR.

**Figure 7:**
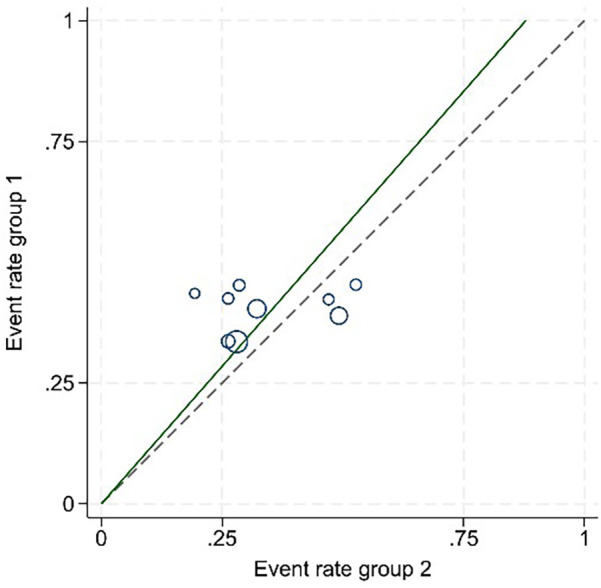
The Label plot.

**Figure 8:**
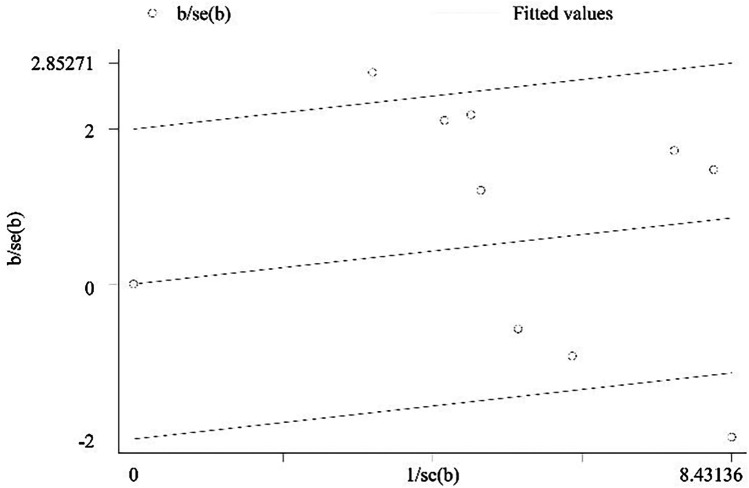
The Galbraith plot.

Following an examination of the heterogeneity of the extant literature (Figure 6), the results of this study demonstrate that *I*^2^ = 68% >50%, and the *P*-value of the *Q* test is 0.001 < 0.1. A more thorough investigation of the Label plot and the Galbraith plot (Figures 7, 8) indicates that this study displays considerable heterogeneity, thereby necessitating subgroup analysis to ascertain the underlying causes of this heterogeneity.

### Subgroup analysis

For the outcome of clinical pregnancy rate, significant heterogeneity was observed across studies (*I*^2^ = 68%, *P* = 0.001), so a random-effect model was used for meta-analysis.

#### Treatments in the control groups

To explore the source of heterogeneity, we conducted subgroup analysis stratified by “treatments in the control groups”: heterogeneity decreased to *I*^2^ = 30.6% (*P* = 0.173), suggesting that treatments in the control groups might be a key factor contributing to the initial heterogeneity.

It was observed that the blank control group exhibited an *I*^2^ of 11.6%, which is less than 50%, with a *Q* test *P*-value of 0.340 that is greater than 0.1. Similarly, the sham acupuncture group demonstrated an *I*^2^ of 20.7%, which is also less than 50%, with a *Q* test *P*-value of 0.286 that is greater than 0.1.

**Figure 9:**
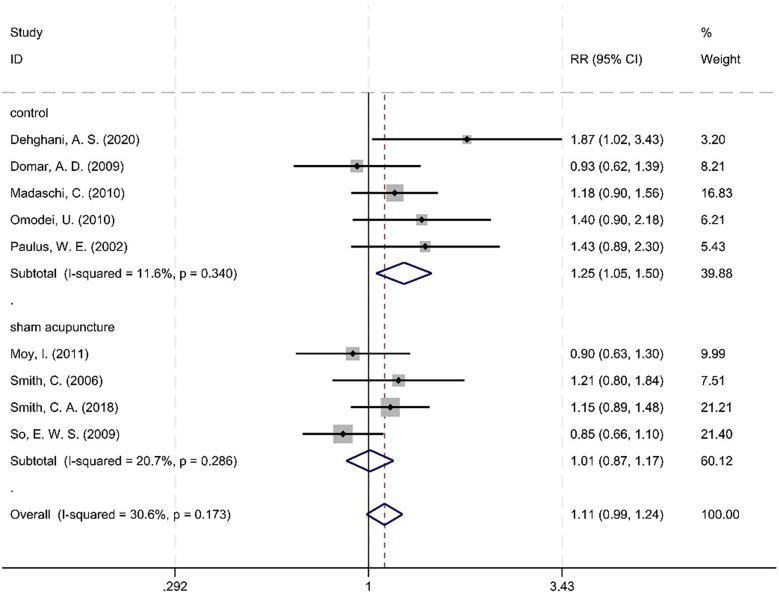
Meta-analysis forest plot between the acupuncture group and the sham acupuncture group.

Subsequent subgroup analysis revealed the absence of heterogeneity among the studies. The pooled RR value for the five studies in the blank control group was 1.25, with a 95% CI of 1.05–1.50, *Z* = 2.50, *P* = 0.013 < 0.05, indicating a statistically significant difference between the acupuncture group and the blank control group. The RR value of the sham acupuncture group in the summary of four literature reports was 1.01, with a 95% CI (0.87–1.17), *Z* = 0.12, *P* = 0.907 > 0.05, indicating that there was no statistically significant difference between the acupuncture group and the sham acupuncture group (Figure 9).

A statistically significant interaction effect was detected for the “blank vs. sham control” subgroup comparison (*Q* = 6.41, *P* = 0.011), demonstrating that the direction and magnitude of acupuncture's effect on CPR varied by control group.

#### Acupuncture timing

**Figure 10:**
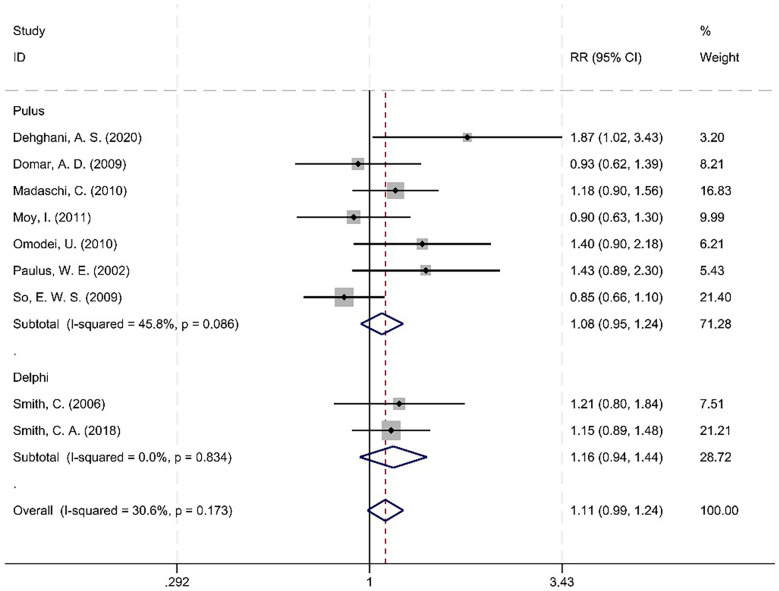
Meta-analysis forest plot between the Pulus Protocol group and the Delphi Consensus group.

It was determined that *I*^2^ = 30.6%, which is less than 50%, and *P* = 0.173, greater than 0.1, indicating an absence of heterogeneity between the studies. The RR value summarized from the seven documents in the Pulus Protocol group was 1.083, with a 95% CI of (0.946–1.240), *Z* = 1.16, *P* = 0.247 > 0.05. The RR value of the two literature summaries of the Delphi Consensus was 1.164, with a 95% CI of (0.938–1.445), *Z* = 1.38, *P* = 0.167 > 0.05. A subsequent analysis revealed no statistically significant difference in CPR between the Pulus Protocol and Delphi Consensus groups (Figure 10).

No statistically significant interaction effect was observed between the subgroup of the acupuncture timing (*Q* = 3.24, *P* = 0.073), indicating that the effect of acupuncture on CPR did not differ substantially between Pulus Protocol and Delphi Consensus groups.

While meta-regression and subgroup analyses identified maternal treatments in the control groups and acupuncture timing as key heterogeneity sources, 30.6% of residual heterogeneity in clinical pregnancy rate remains unexplained. This may stem from unreported variables (e.g., age, acupuncturist qualification) or random variation, highlighting the need for standardized reporting of acupuncture protocols in future IVF trials.

### Publication bias

**Figure 11:**
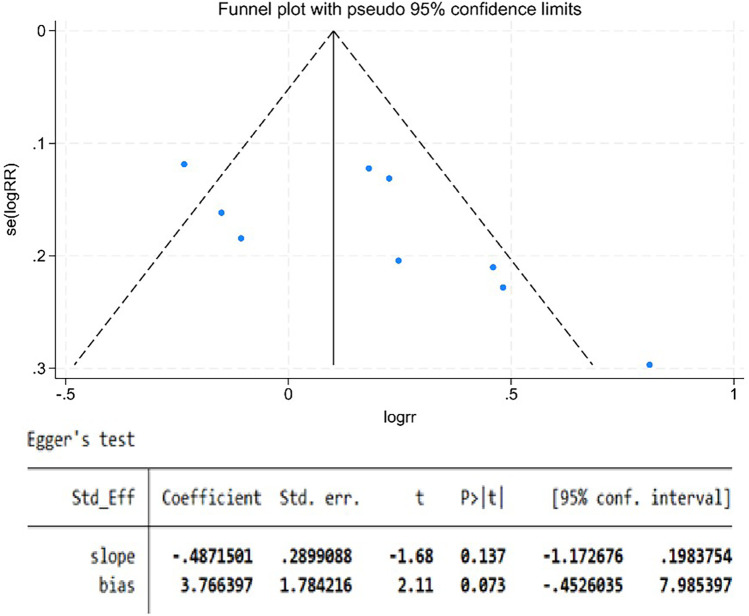
The funnel plot.

Publication bias was assessed using Egger's linear regression test, which returned a *P*-value of 0.073. Importantly, this result requires cautious interpretation due to the limited number of included studies (<10 studies per contrast), a factor that substantially constrains the test's statistical power to detect true asymmetry. Methodological evidence indicates that with fewer than 10 studies per comparison, Egger's test exhibits reduced sensitivity to publication bias and an elevated risk of type II errors. Consequently, the near-significant *P*-value (0.073) should not be interpreted as indicative of publication bias; rather, it represents an inconclusive finding given the constraints of our dataset (Figure 11).

### Secondary outcome

Table 4 summarizes key findings for CPR and LBR—critical reproductive outcomes—presenting comparative effects between intervention and control groups. It includes the number of contributing studies (9 for CPR; 4 for LBR), total sample sizes, and pooled effect sizes with 95% confidence intervals [CPR: 1.18 [0.97–1.43]; LBR: 1.01 [0.88–1.15]]. Heterogeneity, measured via *I*^2^, was moderate-to-high for CPR (68%) and moderate for LBR (40%). GRADE evidence quality ratings are also included: CPR was rated low, primarily due to substantial heterogeneity and imprecision, while LBR was moderate, with downgrading limited to imprecision. This summary concisely conveys effect magnitudes, evidence strength, and cross-study consistency, facilitating rapid interpretation of core results.

**Table 4:** Finding table.

Outcome	Number of studies	Sample size (A/C)	Effect size (95% CI)	Evidence 1uality (GRADE)
CPR	9	1,194/1,186	1.18 (0.97–1.43)	Low
LBR	4	1,222/1,221	1.01 (0.88–1.15)	Moderate

**Figure 12:**
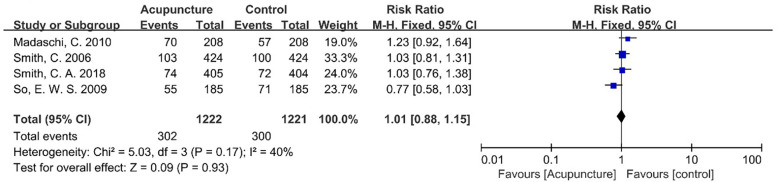
Meta-analysis forest plot for LBR.

The literature reviewed in this study underwent heterogeneity testing, with an *I*^2^ value of 50% and a *Q* test *P*-value of 0.17, indicating that there was no heterogeneity among the selected literature. The *Z*-value was found to be 0.09, and the *P*-value was determined to be 0.93, which is greater than 0.05. This indicates that there is no statistically significant difference in the LBR between the acupuncture group and the control group (Figure 12).

To estimate the risk of type II error (i.e., failure to detect a true effect due to inadequate sample size), *post hoc* power calculations were performed using Stata (via the metapower command), with parameters aligned to consensus standards in IVF research: for the observed effect size (RR = 1.01), the achieved power was 5.8%—substantially below the conventional 80% threshold. This low power is clinically irrelevant, however, as the 0.14% absolute difference in live birth rate is too small to have practical implications for IVF practice.

## Discussion

Results of the meta-analysis, incorporating 11 studies with a total of 3,561 women, provided support for our hypothesis that acupuncture exerts a favorable influence on the success of clinical pregnancy outcomes in ET procedures, the impact of acupuncture on the LBR in ET remains to be elucidated.

The day of embryo transfer represents a time-critical window for optimizing implantation success, as it coincides with the transition of the embryo from the cleavage stage to the blastocyst stage, as well as its first direct interaction with the endometrial lining. This event is dependent on precise synchronization between embryonic developmental potential and endometrial receptivity, a process also known as the “implantation window.” (22)

Evidence suggests that defects during embryo implantation can disrupt gestation, emphasizing its importance (23). A prior study aimed to determine the correlation between endometrial thickness on the day of trigger administration and subsequent outcomes—live birth rate, clinical pregnancy rate, embryo implantation rate, and miscarriage rate—in fresh embryo transfer cycles (24).

Norepinephrine (NE), a key neurotransmitter that modulates hypothalamic neuronal activity, is a potential stimulator of gonadotropin-releasing hormone (GnRH) release from the hypothalamus (25). Investigators of prior studies reported that women with a successful pregnancy outcome had lower adrenaline concentrations at oocyte retrieval and lower concentrations of adrenaline and NE at ET, relative to counterparts with an unsuccessful pregnancy outcome (26, 27).

In the original study (28), serum NE levels were elevated on day 1; notably, compared with untreated rats, acupuncture -treated rats exhibited a significant reduction in serum NE levels on days 7 and 13. The investigators concluded that acupuncture could modulate the homeostasis of the hypothalamic-pituitary-ovarian (HPO) axis in physiologically normal rats.

The practice of acupuncture has been demonstrated to enhance uterine blood supply, reduce uterine contractions, promote embryo implantation, and alleviate stress, tension, anxiety, and depression. Consequently, it has been shown to improve IVF pregnancy outcomes (29, 30).

It is hypothesized that acupuncture could enhance endometrial receptivity by modifying neuroendocrinological factors, increasing uterine blood flow and modulating immune responses (31, 32). The meta-analysis offers an overview of the meridians SP, with a particular focus on their utilization. It is noteworthy that the most prevalent point is SP6.The majority of scholars concur that the distribution of the nerve segments of the uterus and SP6 is analogous, or at least comparable. Consequently, clinically, the method of utilizing SP6 to treat obstetrics and gynecology diseases related to the uterus is a valid approach (33). SP6 is effective in improving blood flow in uterine arteries by reducing uterine artery resistance index (RI) (34).

Sham acupuncture is intended to isolate the effect of needling itself, which has driven the development of appropriate control procedures—including nonpenetrating needles, superficial needling, and needles inserted at nonacupuncture points (35).

In RCTs evaluating the efficacy of acupuncture, it is imperative to establish a physiologically inert control that has the potential to serve as a true placebo. The optimal sham acupuncture configuration must be physiologically inert and indistinguishable from authentic acupuncture. In the course of their research (7, 13, 15, 18, 20), the investigators established a sham acupuncture control group. However, they did not demonstrate that the sham acupuncture was physiologically inert, despite it generally being different from acupuncture. The potential exists for sham contact with acupuncture needles to be a modified form of acupuncture, instead of a true placebo effect (36).

The efficacy of acupuncture and sham acupuncture varies according to the dermatomes stimulated, demonstrating significant overlap in cases of high overlap, and marked differences in cases of low overlap. In order to conduct a controlled trial of acupuncture vs. sham acupuncture, the chosen sites for the application of the latter should be located on non-overlapping dermatomes (37).

It is noteworthy that there are articles reporting different types of sham acupuncture and that these have found, when compared against sham trials with penetrating needles, lower effect sizes for acupuncture than trials with non-penetrating needles (38). The question of the most suitable control group for acupuncture trials remains a matter of debate. This is due to the recognized fact that nonpenetrating and superficial acupuncture needle techniques do not constitute complete inertness (39).

Furthermore, the psychological effects of sham acupuncture cannot be disregarded. Notwithstanding the impact of anxiety on IVF birth outcomes, patients undergoing the process frequently experience high levels of anxiety. Acupuncture aimed at alleviating anxiety has clinical effects with good compliance (40). It is a commonly held belief that study participants will hold the conviction that they have received an effective treatment. Anxiety is a subjective symptom, which means that its treatment can be problematic. It is possible to induce a placebo effect during treatment by using a highly recognized treatment (41).

In subsequent RCT designs, three distinct groupings can be configured: namely, a control group without intervention, an acupuncture group and a sham acupuncture group. The distinction between the non-specific effects of acupuncture and those of sham acupuncture can be minimized to the greatest extent by employing the effective blind and random method.

Presently, the most widely implemented acupuncture protocol in the context of IVF is that which was established by Pulus et al. in 2002. This protocol entails the administration of 25 min of acupuncture treatment 25 min prior to and following ET. The Delphi Consensus (42) is predicated on the scheme proposed by Pulus Protocol, with modifications, namely the incorporation of one acupuncture session on days 6–8 of the ovulation induction cycle, with needles retained for a duration of 25 min.

It has been established that the intensity of acupuncture stimulation is a primary factor influencing the efficacy of acupuncture therapy. The precise understanding and exploration of the dose-response relationship have consistently been pivotal domains of focus in the sphere of clinical research. Furthermore, there is a strong relationship between the time at which acupuncture is administered and its effectiveness. The meta-analysis concluded that no statistically significant difference was identified between Delphi consensus and D Protocol in relation to CPR. Consequently, it can be hypothesized that the acupuncture treatment delivered on days 6–8 of the ovulation induction cycle was not efficacious in enhancing follicle quality.

Acupuncture necessitates a comparatively protracted intervention period to enhance ovarian function, such as the entirety of an IVF cycle or three menstrual cycles in advance (43). The Delphi Consensus guidelines recommend the administration of acupuncture once during the ovulation induction period, specifically on days 6–8. The duration of needle retention is advised to be 25 min. It remains to be ascertained whether this level of stimulation is adequate to promote the maturation of ova and the formation of the corpus luteum. Further discussion and exploration are necessary to determine this.

The window of implantation, as previously defined, is characterized as a narrow time frame of maximal endometrial receptivity, thereby enabling the endometrium to provide a suitable environment for optimal embryo development and placenta formation (44).

A negative correlation was identified between the frequency of uterine contractions immediately prior to the ET procedure and the implantation and CPR (45). In the United States, 44% of infertile women undergoing IVF-ET administrate acupuncture (46). Acupuncture might be beneficial in women undergoing IVF-ET by increasing endometrial blood flow and endometrial receptivity.

The findings of this meta-analysis indicate that the utilization of acupuncture during ET demonstrates a favorable impact on CPR, though no such influence is observed on the LBR. The underlying reason for the observed discrepancy between the enhancement in CPR and the unimproved the LBR is attributable to the fact that the processes of embryo implantation and pregnancy maintenance are governed by disparate biological mechanisms. Furthermore, the requisite sample size for determining CPR is comparatively modest. This facilitates the detection of favorable outcomes; however, the determination of the LBR necessitates a more substantial sample size due to their reduced incidence. Absence of statistical power can impede this process if this size is not adequately met.

Successful embryo implantation relies on effective dialogue between an endometrium in a receptive state and an embryo with adequate developmental competence. Both the receptive endometrium and the developmentally competent embryo secrete extracellular exosomes, which package essential signaling cues required for successful embryo implantation (47). Under a favorable microenvironment orchestrated by steroid hormones, anti-apoptotic mechanisms, and energy-generating pathways, this signal exchange induces cell adhesion and migration, thereby enabling synchronized embryo-endometrial coordination. Endometrial thickness is possibly the most widely used marker for determining endometrial receptivity.

A retrospective study showed that the endometrial thickness was not predictive for live birth in either fresh or frozen-thawed ET cycles (48). In fresh IVF cycles, an increase in endometrial thickness was associated with significantly higher mean numbers of retrieved oocytes, mean peak estradiol concentrations, and mean numbers of usable embryos (49). The LBR is contingent upon the successful maintenance of the pregnancy, a process which is protracted over time.

Miscarriage is generally defined as the loss of a pregnancy before viability (50). Both miscarriage and, more notably, recurrent miscarriage act as sentinel risk markers for obstetric complications in future pregnancies (e.g., preterm birth, fetal growth restriction, placental abruption, stillbirth) and as predictors of long-term health issues. Chromosomal errors, uterine anatomical anomalies, autoimmune conditions, and endometrial dysfunction are established etiological factors contributing to recurrent pregnancy loss (51). Establishing an immune tolerance microenvironment and abundant blood supply are the prerequisites for pregnancy maintenance (52). Simultaneously, embryo quality has been identified as the primary factor influencing the cumulative LBR subsequent to elective single ET in fresh stimulation cycles (53). Consequently, the potential of acupuncture to enhance the LBR of IVF-ET remains debatable (54).

Embryo transfer is also associated with factors related to previous pregnancy history. A decline in biochemical pregnancy and live birth rates has been observed in patients with a prior cesarean section (CS) compared to vaginal delivery (55). A meta-analysis of observational studies (including 13,696 infertile women) demonstrated that, compared with women with a history of vaginal delivery, those with a prior CS had significantly lower biochemical pregnancy rates and LBR (56).

Differences in maternal and neonatal outcomes may be attributed to three key factors: inherent disparities in groups assigned to different endometrial preparation types, supraphysiologic hormone environments in programmed cycles, and the lack of unique corpus luteum secretions (characteristic of ovulatory cycles) in programmed cycles (57).

Ovarian stimulation methodologies varied across the included randomized controlled trials. Within assisted reproductive technology, the most commonly employed ovarian stimulation protocols include the long protocol, antagonist protocol, progestin-primed ovarian stimulation protocol, and mild stimulation protocol. Among these, the included studies utilized the long protocol and antagonist protocol exclusively. Notably, the process known as ovulation induction has been observed to potentially impair the functionality of the endometrial tissue due to insufficient progesterone secretion (58). This phenomenon is of particular concern, given that a paucity of progesterone has been demonstrated to be associated with adverse pregnancy outcomes, as evidenced by research. Consequently, in subsequent randomized controlled trials, the selection of ovarian stimulation protocols must be standardized to mitigate the impact of superovulation protocols on hormones and the uterus.

A notable limitation of the studies included herein is the omission of discussion regarding additional confounding factors modulating CPR/LBR in ART that have been shown to influence endometrial receptivity. Multiple studies (59) have shown a potential correlation between chronic endometritis (CE) and reproductive disorders. The detrimental impact of CE on fertility is often attributed to the aberrant infiltration of plasma cells (60), which subsequently lead to the release of antibodies and cytokines.

Despite the novel insights this work adds to the existing evidence base regarding acupuncture as an adjunctive therapy on embryo transfer day, our study is not without limitations that merit careful consideration when contextualizing and interpreting our findings.

A key limitation among the included RCTs is the absence of adverse event reporting—including rare occurrences—and data on participant withdrawals linked to adverse events in six studies. This aligns with broader reproductive medicine research observations, where safety outcomes—especially rare or non-serious ones—are consistently underreported relative to efficacy endpoints like CPR or LBR. Future RCTs in this field should prioritize standardized reporting of adverse events (including rare but clinically meaningful cases), which is critical to refining evidence-based clinical decisions and ensuring balanced understanding of intervention safety and efficacy.

A methodological limitation that must be acknowledged pertains to the reduced statistical power of Egger's test for assessing publication bias. This limitation arises from the inclusion of fewer than 10 studies per contrast. This constraint impairs our capacity to definitively evaluate potential small-study effects, as methodological consensus indicates that statistical tests for publication bias—including Egger's test—exhibit suboptimal performance when the number of studies per comparison is this limited.

A potential limitation is our restriction to English-language studies—this decision prioritized data extraction accuracy, given the team's limited non-English proficiency and lack of dedicated translation support, as linguistic barriers risk interpretive bias. While this minimizes error, it may exclude relevant non-English research; future reviews with multilingual teams or certified translation resources could address this gap for a more comprehensive global synthesis.

In clinical studies of acupuncture intervention for ET, the effect of the acupuncture regimen on endometrial receptivity should first be assessed, followed by an extension of the study duration to evaluate the endpoint criteria. In the context of future acupuncture-assisted IVF studies, it is imperative that research methodologies adhere meticulously to the requirements of evidence-based medicine. Such adherence is crucial for enhancing the quality of acupuncture-assisted IVF clinical research and ensuring the reliability of its conclusions. At present, there is a paucity of sham acupuncture control designs that can adequately differentiate the placebo effect of acupuncture without introducing other risk biases. In order to achieve a balanced and unbiased assessment of the clinical effectiveness of acupuncture in IVF applications, the establishment of three control groups is essential. These groups should include an acupuncture group, a blank control group, and a sham acupuncture group, thereby enhancing the credibility of the study and ensuring the robustness of the results.

## Conclusion

Our meta-analysis of acupuncture in ET reveals context-dependent outcomes: pooled data suggest a potential benefit of acupuncture for CPR when compared to blank control, yet no advantage is observed for CPR vs. sham acupuncture or for LBR—the primary endpoint—across all control types. Future RCT should prioritize methodologically rigorous sham-controlled designs, such as the adoption of validated blinding protocols, to reduce bias. Additional critical evidence gaps include the lack of data on long-term neonatal outcomes and mechanistic markers, which are essential to clarifying acupuncture's safety profile and biological mechanisms in assisted reproductive settings. Addressing these priorities will facilitate more robust assessments of acupuncture's efficacy in ET for subsequent research.

## Data Availability

The original contributions presented in the study are included in the article/Supplementary Material, further inquiries can be directed to the corresponding author.
