# Supplementary material for: Acupuncture as an adjunctive therapy on embryo transfer day: a systematic review and meta-analysis of clinical pregnancy and live birth outcomes
Source: Front Reprod Health. 2025 Sep 23;7:1673144. doi: 10.3389/frph.2025.1673144 (PMC12500596; doi:10.3389/frph.2025.1673144)
Supplement: Supplementary file 1 [file Datasheet1.docx]

Search number,Query,Sort By,Filters,Search Details,Results,Time

37,"((((((""Embryo Transfer""[Mesh]) OR ((((((Embryo Transfers[Title/Abstract]) OR (Transfer, Embryo[Title/Abstract])) OR (Transfers, Embryo[Title/Abstract])) OR (Tubal Embryo Transfer[Title/Abstract])) OR (Tubal Embryo Stage Transfer[Title/Abstract])) OR (Blastocyst Transfer[Title/Abstract]))) OR ((""Reproductive Techniques, Assisted""[Mesh]) OR (((((((((((((((((Assisted Reproductive Technique[Title/Abstract]) OR (Reproductive Technique, Assisted[Title/Abstract])) OR (Technique, Assisted Reproductive[Title/Abstract])) OR (Techniques, Assisted Reproductive[Title/Abstract])) OR (Assisted Reproductive Technics[Title/Abstract])) OR (Assisted Reproductive Technic[Title/Abstract])) OR (Reproductive Technic, Assisted[Title/Abstract])) OR (Reproductive Technics, Assisted[Title/Abstract])) OR (Technic, Assisted Reproductive[Title/Abstract])) OR (Technics, Assisted Reproductive[Title/Abstract])) OR (Assisted Reproductive Techniques[Title/Abstract])) OR (Reproductive Technology, Assisted[Title/Abstract])) OR (Assisted Reproductive Technologies[Title/Abstract])) OR (Assisted Reproductive Technology[Title/Abstract])) OR (Reproductive Technologies, Assisted[Title/Abstract])) OR (Technologies, Assisted Reproductive[Title/Abstract])) OR (Technology, Assisted Reproductive[Title/Abstract])))) OR ((""Fertilization in Vitro""[Mesh]) OR (((((((((((((In Vitro Fertilization[Title/Abstract]) OR (In Vitro Fertilizations[Title/Abstract])) OR (Test-Tube Fertilization[Title/Abstract])) OR (Fertilizations, Test-Tube[Title/Abstract])) OR (Fertilization, Test-Tube[Title/Abstract])) OR (Test Tube Fertilization[Title/Abstract])) OR (Test-Tube Fertilizations[Title/Abstract])) OR (Fertilizations in Vitro[Title/Abstract])) OR (Test-Tube Babies[Title/Abstract])) OR (Babies, Test-Tube[Title/Abstract])) OR (Baby, Test-Tube[Title/Abstract])) OR (Test Tube Babies[Title/Abstract])) OR (Test-Tube Baby[Title/Abstract])))) OR ((""Oocytes""[Mesh]) OR (((Oocyte[Title/Abstract]) OR (Ovocytes[Title/Abstract])) OR (Ovocyte[Title/Abstract])))) AND ((((((((""Acupuncture""[Mesh]) OR (Pharmacopuncture[Title/Abstract])) OR ((""Acupuncture Therapy""[Mesh]) OR ((((((((((Acupuncture Treatment[Title/Abstract]) OR (Acupuncture Treatments[Title/Abstract])) OR (Treatment, Acupuncture[Title/Abstract])) OR (Therapy, Acupuncture[Title/Abstract])) OR (Pharmacoacupuncture Treatment[Title/Abstract])) OR (Treatment, Pharmacoacupuncture[Title/Abstract])) OR (Pharmacoacupuncture Therapy[Title/Abstract])) OR (Therapy, Pharmacoacupuncture[Title/Abstract])) OR (Acupotomy[Title/Abstract])) OR (Acupotomies[Title/Abstract])))) OR ((""Auriculotherapy""[Mesh]) OR (Auriculotherapies[Title/Abstract]))) OR ((""Moxibustion""[Mesh]) OR (Moxabustion[Title/Abstract]))) OR ((""Medicine, East Asian Traditional""[Mesh]) OR ((((((((((((((((((((((((((((Oriental Traditional Medicine[Title/Abstract]) OR (Oriental Medicine, Traditional[Title/Abstract])) OR (Medicine, Traditional Oriental[Title/Abstract])) OR (Traditional Oriental Medicine[Title/Abstract])) OR (Traditional Oriental Medicines[Title/Abstract])) OR (Traditional Medicine, Oriental[Title/Abstract])) OR (Medicine, Oriental Traditional[Title/Abstract])) OR (Medicine, Traditional, East Asia[Title/Abstract])) OR (Traditional Medicine, East Asia[Title/Abstract])) OR (Traditional Far Eastern Medicine[Title/Abstract])) OR (East Asian Traditional Medicine[Title/Abstract])) OR (Traditional East Asian Medicine[Title/Abstract])) OR (East Asian Medicine[Title/Abstract])) OR (East Asian Medicines[Title/Abstract])) OR (Medicine, East Asian[Title/Abstract])) OR (Medicine, Oriental[Title/Abstract])) OR (Oriental Medicine[Title/Abstract])) OR (Medicine, East Asia[Title/Abstract])) OR (Asia Medicines, East[Title/Abstract])) OR (East Asia Medicine[Title/Abstract])) OR (East Asia Medicines[Title/Abstract])) OR (Medicines, East Asia[Title/Abstract])) OR (Medicine, Far East[Title/Abstract])) OR (East Medicine, Far[Title/Abstract])) OR (East Medicines, Far[Title/Abstract])) OR (Far East Medicine[Title/Abstract])) OR (Far East Medicines[Title/Abstract])) OR (Medicines, Far East[Title/Abstract])))) OR ((""Medicine, Chinese Traditional""[Mesh]) OR ((((((((((((((Zhong Yi Xue[Title/Abstract]) OR (Chung I Hsueh[Title/Abstract])) OR (Hsueh, Chung I[Title/Abstract])) OR (Traditional Medicine, Chinese[Title/Abstract])) OR (Chinese Traditional Medicine[Title/Abstract])) OR (Traditional Chinese Medicine[Title/Abstract])) OR (Chinese Medicine, Traditional[Title/Abstract])) OR (Traditional Tongue Diagnosis[Title/Abstract])) OR (Tongue Diagnoses, Traditional[Title/Abstract])) OR (Tongue Diagnosis, Traditional[Title/Abstract])) OR (Traditional Tongue Diagnoses[Title/Abstract])) OR (Traditional Tongue Assessment[Title/Abstract])) OR (Tongue Assessment, Traditional[Title/Abstract])) OR (Traditional Tongue Assessments[Title/Abstract])))) OR (""Electroacupuncture""[Mesh]))) AND ((""Randomized Controlled Trial"" [Publication Type]) OR ((Randomized[Title/Abstract]) OR (Placebo[Title/Abstract])))",,,"(""Embryo Transfer""[MeSH Terms] OR (""embryo transfers""[Title/Abstract] OR ""transfer embryo""[Title/Abstract] OR ""transfers embryo""[Title/Abstract] OR ""tubal embryo transfer""[Title/Abstract] OR ""tubal embryo stage transfer""[Title/Abstract] OR ""blastocyst transfer""[Title/Abstract]) OR (""reproductive techniques, assisted""[MeSH Terms] OR (""assisted reproductive technique""[Title/Abstract] OR ""reproductive technique assisted""[Title/Abstract] OR ((""methods""[MeSH Terms] OR ""methods""[All Fields] OR ""Technique""[All Fields] OR ""methods""[MeSH Subheading] OR ""Techniques""[All Fields] OR ""technique s""[All Fields]) AND ""assisted reproductive""[Title/Abstract]) OR ((""methods""[MeSH Terms] OR ""methods""[All Fields] OR ""Technique""[All Fields] OR ""methods""[MeSH Subheading] OR ""Techniques""[All Fields] OR ""technique s""[All Fields]) AND ""assisted reproductive""[Title/Abstract]) OR ""assisted reproductive technics""[Title/Abstract] OR ""assisted reproductive technic""[Title/Abstract] OR (((""reproduction""[MeSH Terms] OR ""reproduction""[All Fields] OR ""reproductions""[All Fields] OR ""Reproductive""[All Fields] OR ""reproductively""[All Fields] OR ""reproductives""[All Fields] OR ""reproductivity""[All Fields]) AND (""Technic""[All Fields] OR ""Technics""[All Fields])) AND ""Assisted""[Title/Abstract]) OR (((""reproduction""[MeSH Terms] OR ""reproduction""[All Fields] OR ""reproductions""[All Fields] OR ""Reproductive""[All Fields] OR ""reproductively""[All Fields] OR ""reproductives""[All Fields] OR ""reproductivity""[All Fields]) AND (""Technic""[All Fields] OR ""Technics""[All Fields])) AND ""Assisted""[Title/Abstract]) OR ((""Technic""[All Fields] OR ""Technics""[All Fields]) AND ""assisted reproductive""[Title/Abstract]) OR ((""Technic""[All Fields] OR ""Technics""[All Fields]) AND ""assisted reproductive""[Title/Abstract]) OR ""assisted reproductive techniques""[Title/Abstract] OR ((""reproduction""[MeSH Terms] OR ""reproduction""[All Fields] OR ""reproductions""[All Fields] OR ""Reproductive""[All Fields] OR ""reproductively""[All Fields] OR ""reproductives""[All Fields] OR ""reproductivity""[All Fields]) AND ""technology assisted""[Title/Abstract]) OR ""assisted reproductive technologies""[Title/Abstract] OR ""assisted reproductive technology""[Title/Abstract] OR ""reproductive technologies assisted""[Title/Abstract] OR ""technologies assisted reproductive""[Title/Abstract] OR ""technology assisted reproductive""[Title/Abstract])) OR (""Fertilization in Vitro""[MeSH Terms] OR (""in vitro fertilization""[Title/Abstract] OR ""in vitro fertilizations""[Title/Abstract] OR ""test tube fertilization""[Title/Abstract] OR ((""fertilisability""[All Fields] OR ""fertilisable""[All Fields] OR ""fertilisation""[All Fields] OR ""Fertilization""[MeSH Terms] OR ""Fertilization""[All Fields] OR ""fertilisations""[All Fields] OR ""fertilise""[All Fields] OR ""fertilised""[All Fields] OR ""fertilisers""[All Fields] OR ""fertilizers""[Pharmacological Action] OR ""fertilizers""[Supplementary Concept] OR ""fertilizers""[All Fields] OR ""fertilizers""[MeSH Terms] OR ""fertilises""[All Fields] OR ""fertilising""[All Fields] OR ""Fertilizations""[All Fields] OR ""fertilize""[All Fields] OR ""fertilized""[All Fields] OR ""fertiliser""[All Fields] OR ""fertilizer""[All Fields] OR ""fertilizes""[All Fields] OR ""fertilizing""[All Fields]) AND ""Test-Tube""[Title/Abstract]) OR ""fertilization test tube""[Title/Abstract] OR ""test tube fertilization""[Title/Abstract] OR (""Test-Tube""[All Fields] AND ""Fertilizations""[Title/Abstract]) OR ""fertilizations in vitro""[Title/Abstract] OR ""test tube babies""[Title/Abstract] OR ((""baby s""[All Fields] OR ""babys""[All Fields] OR ""infant""[MeSH Terms] OR ""infant""[All Fields] OR ""Babies""[All Fields]) AND ""Test-Tube""[Title/Abstract]) OR ((""infant, newborn""[MeSH Terms] OR (""infant""[All Fields] AND ""newborn""[All Fields]) OR ""newborn infant""[All Fields] OR ""Baby""[All Fields] OR ""infant""[MeSH Terms] OR ""infant""[All Fields]) AND ""Test-Tube""[Title/Abstract]) OR ""test tube babies""[Title/Abstract] OR ""test tube baby""[Title/Abstract])) OR (""Oocytes""[MeSH Terms] OR (""Oocyte""[Title/Abstract] OR ""Ovocytes""[Title/Abstract] OR ""Ovocyte""[Title/Abstract]))) AND (""Acupuncture""[MeSH Terms] OR ""Pharmacopuncture""[Title/Abstract] OR (""Acupuncture Therapy""[MeSH Terms] OR (""acupuncture treatment""[Title/Abstract] OR ""acupuncture treatments""[Title/Abstract] OR ""treatment acupuncture""[Title/Abstract] OR ""therapy acupuncture""[Title/Abstract] OR ""pharmacoacupuncture treatment""[Title/Abstract] OR ((""therapeutics""[MeSH Terms] OR ""therapeutics""[All Fields] OR ""Treatments""[All Fields] OR ""Therapy""[MeSH Subheading] OR ""Therapy""[All Fields] OR ""Treatment""[All Fields] OR ""treatment s""[All Fields]) AND ""Pharmacoacupuncture""[Title/Abstract]) OR ""pharmacoacupuncture therapy""[Title/Abstract] OR ((""therapeutics""[MeSH Terms] OR ""therapeutics""[All Fields] OR ""therapies""[All Fields] OR ""Therapy""[MeSH Subheading] OR ""Therapy""[All Fields] OR ""therapy s""[All Fields] OR ""therapys""[All Fields]) AND ""Pharmacoacupuncture""[Title/Abstract]) OR ""Acupotomy""[Title/Abstract] OR ""Acupotomies""[Title/Abstract])) OR (""Auriculotherapy""[MeSH Terms] OR ""Auriculotherapies""[Title/Abstract]) OR (""Moxibustion""[MeSH Terms] OR ""Moxabustion""[Title/Abstract]) OR (""medicine, east asian traditional""[MeSH Terms] OR (""oriental traditional medicine""[Title/Abstract] OR ((""asian people""[MeSH Terms] OR (""Asian""[All Fields] AND ""people""[All Fields]) OR ""asian people""[All Fields] OR ""Oriental""[All Fields] OR ""orientals""[All Fields]) AND ""medicine traditional""[Title/Abstract]) OR ((""medicin""[All Fields] OR ""medicinal""[All Fields] OR ""medicinally""[All Fields] OR ""medicinals""[All Fields] OR ""Medicine""[MeSH Terms] OR ""Medicine""[All Fields] OR ""medicine s""[All Fields] OR ""Medicines""[All Fields]) AND ""traditional oriental""[Title/Abstract]) OR ""traditional oriental medicine""[Title/Abstract] OR ""traditional oriental medicines""[Title/Abstract] OR ""traditional medicine oriental""[Title/Abstract] OR ((""medicin""[All Fields] OR ""medicinal""[All Fields] OR ""medicinally""[All Fields] OR ""medicinals""[All Fields] OR ""Medicine""[MeSH Terms] OR ""Medicine""[All Fields] OR ""medicine s""[All Fields] OR ""Medicines""[All Fields]) AND ""oriental traditional""[Title/Abstract]) OR ((""medicine, traditional""[MeSH Terms] OR (""Medicine""[All Fields] AND ""Traditional""[All Fields]) OR ""traditional medicine""[All Fields] OR ""medicine traditional""[All Fields]) AND ""east asia""[Title/Abstract]) OR ((""tradition""[All Fields] OR ""tradition s""[All Fields] OR ""Traditional""[All Fields] OR ""traditionals""[All Fields] OR ""traditions""[All Fields]) AND ""medicine east asia""[Title/Abstract]) OR ((""tradition""[All Fields] OR ""tradition s""[All Fields] OR ""Traditional""[All Fields] OR ""traditionals""[All Fields] OR ""traditions""[All Fields]) AND ""far eastern medicine""[Title/Abstract]) OR ""east asian traditional medicine""[Title/Abstract] OR ""traditional east asian medicine""[Title/Abstract] OR ""east asian medicine""[Title/Abstract] OR ""east asian medicines""[Title/Abstract] OR ""medicine east asian""[Title/Abstract] OR ""medicine oriental""[Title/Abstract] OR ""oriental medicine""[Title/Abstract] OR ""medicine east asia""[Title/Abstract] OR (((""Asia""[MeSH Terms] OR ""Asia""[All Fields]) AND (""medicin""[All Fields] OR ""medicinal""[All Fields] OR ""medicinally""[All Fields] OR ""medicinals""[All Fields] OR ""Medicine""[MeSH Terms] OR ""Medicine""[All Fields] OR ""medicine s""[All Fields] OR ""Medicines""[All Fields])) AND ""East""[Title/Abstract]) OR ""east asia medicine""[Title/Abstract] OR (""East""[All Fields] AND ""asia medicines""[Title/Abstract]) OR ((""medicin""[All Fields] OR ""medicinal""[All Fields] OR ""medicinally""[All Fields] OR ""medicinals""[All Fields] OR ""Medicine""[MeSH Terms] OR ""Medicine""[All Fields] OR ""medicine s""[All Fields] OR ""Medicines""[All Fields]) AND ""east asia""[Title/Abstract]) OR ((""medicin""[All Fields] OR ""medicinal""[All Fields] OR ""medicinally""[All Fields] OR ""medicinals""[All Fields] OR ""Medicine""[MeSH Terms] OR ""Medicine""[All Fields] OR ""medicine s""[All Fields] OR ""Medicines""[All Fields]) AND ""far east""[Title/Abstract]) OR (""East""[All Fields] AND ""medicine far""[Title/Abstract]) OR ((""East""[All Fields] AND (""medicin""[All Fields] OR ""medicinal""[All Fields] OR ""medicinally""[All Fields] OR ""medicinals""[All Fields] OR ""Medicine""[MeSH Terms] OR ""Medicine""[All Fields] OR ""medicine s""[All Fields] OR ""Medicines""[All Fields])) AND ""Far""[Title/Abstract]) OR ""far east medicine""[Title/Abstract] OR ""far east medicines""[Title/Abstract] OR ((""medicin""[All Fields] OR ""medicinal""[All Fields] OR ""medicinally""[All Fields] OR ""medicinals""[All Fields] OR ""Medicine""[MeSH Terms] OR ""Medicine""[All Fields] OR ""medicine s""[All Fields] OR ""Medicines""[All Fields]) AND ""far east""[Title/Abstract]))) OR (""medicine, chinese traditional""[MeSH Terms] OR (""zhong yi xue""[Title/Abstract] OR ((chung i[Author] OR chung i[Investigator]) AND ""Hsueh""[Title/Abstract]) OR (""Hsueh""[All Fields] AND ""chung i""[Title/Abstract]) OR ""traditional medicine chinese""[Title/Abstract] OR ""chinese traditional medicine""[Title/Abstract] OR ""traditional chinese medicine""[Title/Abstract] OR ""chinese medicine traditional""[Title/Abstract] OR ""traditional tongue diagnosis""[Title/Abstract] OR (((""Tongue""[MeSH Terms] OR ""Tongue""[All Fields] OR ""tongues""[All Fields] OR ""tongue s""[All Fields]) AND (""diagnosable""[All Fields] OR ""diagnosi""[All Fields] OR ""Diagnosis""[MeSH Terms] OR ""Diagnosis""[All Fields] OR ""diagnose""[All Fields] OR ""diagnosed""[All Fields] OR ""Diagnoses""[All Fields] OR ""diagnosing""[All Fields] OR ""Diagnosis""[MeSH Subheading])) AND ""Traditional""[Title/Abstract]) OR (((""Tongue""[MeSH Terms] OR ""Tongue""[All Fields] OR ""tongues""[All Fields] OR ""tongue s""[All Fields]) AND (""diagnosable""[All Fields] OR ""diagnosi""[All Fields] OR ""Diagnosis""[MeSH Terms] OR ""Diagnosis""[All Fields] OR ""diagnose""[All Fields] OR ""diagnosed""[All Fields] OR ""Diagnoses""[All Fields] OR ""diagnosing""[All Fields] OR ""Diagnosis""[MeSH Subheading])) AND ""Traditional""[Title/Abstract]) OR ((""tradition""[All Fields] OR ""tradition s""[All Fields] OR ""Traditional""[All Fields] OR ""traditionals""[All Fields] OR ""traditions""[All Fields]) AND ""tongue diagnoses""[Title/Abstract]) OR ((""tradition""[All Fields] OR ""tradition s""[All Fields] OR ""Traditional""[All Fields] OR ""traditionals""[All Fields] OR ""traditions""[All Fields]) AND ""tongue assessment""[Title/Abstract]) OR ((""Tongue""[MeSH Terms] OR ""Tongue""[All Fields] OR ""tongues""[All Fields] OR ""tongue s""[All Fields]) AND ""assessment traditional""[Title/Abstract]) OR ((""tradition""[All Fields] OR ""tradition s""[All Fields] OR ""Traditional""[All Fields] OR ""traditionals""[All Fields] OR ""traditions""[All Fields]) AND ""tongue assessments""[Title/Abstract]))) OR ""Electroacupuncture""[MeSH Terms]) AND (""Randomized Controlled Trial""[Publication Type] OR (""Randomized""[Title/Abstract] OR ""Placebo""[Title/Abstract]))",193,10:03:41

36,"(""Randomized Controlled Trial"" [Publication Type]) OR ((Randomized[Title/Abstract]) OR (Placebo[Title/Abstract]))",,,"""Randomized Controlled Trial""[Publication Type] OR ""Randomized""[Title/Abstract] OR ""Placebo""[Title/Abstract]","1,123,727",10:02:52

35,(Randomized[Title/Abstract]) OR (Placebo[Title/Abstract]),,,"""Randomized""[Title/Abstract] OR ""Placebo""[Title/Abstract]","891,077",10:02:02

34,"""Randomized Controlled Trial"" [Publication Type]",Most Recent,,"""Randomized Controlled Trial""[Publication Type]","638,915",10:01:36

33,"(((((((""Acupuncture""[Mesh]) OR (Pharmacopuncture[Title/Abstract])) OR ((""Acupuncture Therapy""[Mesh]) OR ((((((((((Acupuncture Treatment[Title/Abstract]) OR (Acupuncture Treatments[Title/Abstract])) OR (Treatment, Acupuncture[Title/Abstract])) OR (Therapy, Acupuncture[Title/Abstract])) OR (Pharmacoacupuncture Treatment[Title/Abstract])) OR (Treatment, Pharmacoacupuncture[Title/Abstract])) OR (Pharmacoacupuncture Therapy[Title/Abstract])) OR (Therapy, Pharmacoacupuncture[Title/Abstract])) OR (Acupotomy[Title/Abstract])) OR (Acupotomies[Title/Abstract])))) OR ((""Auriculotherapy""[Mesh]) OR (Auriculotherapies[Title/Abstract]))) OR ((""Moxibustion""[Mesh]) OR (Moxabustion[Title/Abstract]))) OR ((""Medicine, East Asian Traditional""[Mesh]) OR ((((((((((((((((((((((((((((Oriental Traditional Medicine[Title/Abstract]) OR (Oriental Medicine, Traditional[Title/Abstract])) OR (Medicine, Traditional Oriental[Title/Abstract])) OR (Traditional Oriental Medicine[Title/Abstract])) OR (Traditional Oriental Medicines[Title/Abstract])) OR (Traditional Medicine, Oriental[Title/Abstract])) OR (Medicine, Oriental Traditional[Title/Abstract])) OR (Medicine, Traditional, East Asia[Title/Abstract])) OR (Traditional Medicine, East Asia[Title/Abstract])) OR (Traditional Far Eastern Medicine[Title/Abstract])) OR (East Asian Traditional Medicine[Title/Abstract])) OR (Traditional East Asian Medicine[Title/Abstract])) OR (East Asian Medicine[Title/Abstract])) OR (East Asian Medicines[Title/Abstract])) OR (Medicine, East Asian[Title/Abstract])) OR (Medicine, Oriental[Title/Abstract])) OR (Oriental Medicine[Title/Abstract])) OR (Medicine, East Asia[Title/Abstract])) OR (Asia Medicines, East[Title/Abstract])) OR (East Asia Medicine[Title/Abstract])) OR (East Asia Medicines[Title/Abstract])) OR (Medicines, East Asia[Title/Abstract])) OR (Medicine, Far East[Title/Abstract])) OR (East Medicine, Far[Title/Abstract])) OR (East Medicines, Far[Title/Abstract])) OR (Far East Medicine[Title/Abstract])) OR (Far East Medicines[Title/Abstract])) OR (Medicines, Far East[Title/Abstract])))) OR ((""Medicine, Chinese Traditional""[Mesh]) OR ((((((((((((((Zhong Yi Xue[Title/Abstract]) OR (Chung I Hsueh[Title/Abstract])) OR (Hsueh, Chung I[Title/Abstract])) OR (Traditional Medicine, Chinese[Title/Abstract])) OR (Chinese Traditional Medicine[Title/Abstract])) OR (Traditional Chinese Medicine[Title/Abstract])) OR (Chinese Medicine, Traditional[Title/Abstract])) OR (Traditional Tongue Diagnosis[Title/Abstract])) OR (Tongue Diagnoses, Traditional[Title/Abstract])) OR (Tongue Diagnosis, Traditional[Title/Abstract])) OR (Traditional Tongue Diagnoses[Title/Abstract])) OR (Traditional Tongue Assessment[Title/Abstract])) OR (Tongue Assessment, Traditional[Title/Abstract])) OR (Traditional Tongue Assessments[Title/Abstract])))) OR (""Electroacupuncture""[Mesh])",,,"""Acupuncture""[MeSH Terms] OR ""Pharmacopuncture""[Title/Abstract] OR (""Acupuncture Therapy""[MeSH Terms] OR (""acupuncture treatment""[Title/Abstract] OR ""acupuncture treatments""[Title/Abstract] OR ""treatment acupuncture""[Title/Abstract] OR ""therapy acupuncture""[Title/Abstract] OR ""pharmacoacupuncture treatment""[Title/Abstract] OR ((""therapeutics""[MeSH Terms] OR ""therapeutics""[All Fields] OR ""Treatments""[All Fields] OR ""Therapy""[MeSH Subheading] OR ""Therapy""[All Fields] OR ""Treatment""[All Fields] OR ""treatment s""[All Fields]) AND ""Pharmacoacupuncture""[Title/Abstract]) OR ""pharmacoacupuncture therapy""[Title/Abstract] OR ((""therapeutics""[MeSH Terms] OR ""therapeutics""[All Fields] OR ""therapies""[All Fields] OR ""Therapy""[MeSH Subheading] OR ""Therapy""[All Fields] OR ""therapy s""[All Fields] OR ""therapys""[All Fields]) AND ""Pharmacoacupuncture""[Title/Abstract]) OR ""Acupotomy""[Title/Abstract] OR ""Acupotomies""[Title/Abstract])) OR (""Auriculotherapy""[MeSH Terms] OR ""Auriculotherapies""[Title/Abstract]) OR (""Moxibustion""[MeSH Terms] OR ""Moxabustion""[Title/Abstract]) OR (""medicine, east asian traditional""[MeSH Terms] OR (""oriental traditional medicine""[Title/Abstract] OR ((""asian people""[MeSH Terms] OR (""Asian""[All Fields] AND ""people""[All Fields]) OR ""asian people""[All Fields] OR ""Oriental""[All Fields] OR ""orientals""[All Fields]) AND ""medicine traditional""[Title/Abstract]) OR ((""medicin""[All Fields] OR ""medicinal""[All Fields] OR ""medicinally""[All Fields] OR ""medicinals""[All Fields] OR ""Medicine""[MeSH Terms] OR ""Medicine""[All Fields] OR ""medicine s""[All Fields] OR ""Medicines""[All Fields]) AND ""traditional oriental""[Title/Abstract]) OR ""traditional oriental medicine""[Title/Abstract] OR ""traditional oriental medicines""[Title/Abstract] OR ""traditional medicine oriental""[Title/Abstract] OR ((""medicin""[All Fields] OR ""medicinal""[All Fields] OR ""medicinally""[All Fields] OR ""medicinals""[All Fields] OR ""Medicine""[MeSH Terms] OR ""Medicine""[All Fields] OR ""medicine s""[All Fields] OR ""Medicines""[All Fields]) AND ""oriental traditional""[Title/Abstract]) OR ((""medicine, traditional""[MeSH Terms] OR (""Medicine""[All Fields] AND ""Traditional""[All Fields]) OR ""traditional medicine""[All Fields] OR ""medicine traditional""[All Fields]) AND ""east asia""[Title/Abstract]) OR ((""tradition""[All Fields] OR ""tradition s""[All Fields] OR ""Traditional""[All Fields] OR ""traditionals""[All Fields] OR ""traditions""[All Fields]) AND ""medicine east asia""[Title/Abstract]) OR ((""tradition""[All Fields] OR ""tradition s""[All Fields] OR ""Traditional""[All Fields] OR ""traditionals""[All Fields] OR ""traditions""[All Fields]) AND ""far eastern medicine""[Title/Abstract]) OR ""east asian traditional medicine""[Title/Abstract] OR ""traditional east asian medicine""[Title/Abstract] OR ""east asian medicine""[Title/Abstract] OR ""east asian medicines""[Title/Abstract] OR ""medicine east asian""[Title/Abstract] OR ""medicine oriental""[Title/Abstract] OR ""oriental medicine""[Title/Abstract] OR ""medicine east asia""[Title/Abstract] OR (((""Asia""[MeSH Terms] OR ""Asia""[All Fields]) AND (""medicin""[All Fields] OR ""medicinal""[All Fields] OR ""medicinally""[All Fields] OR ""medicinals""[All Fields] OR ""Medicine""[MeSH Terms] OR ""Medicine""[All Fields] OR ""medicine s""[All Fields] OR ""Medicines""[All Fields])) AND ""East""[Title/Abstract]) OR ""east asia medicine""[Title/Abstract] OR (""East""[All Fields] AND ""asia medicines""[Title/Abstract]) OR ((""medicin""[All Fields] OR ""medicinal""[All Fields] OR ""medicinally""[All Fields] OR ""medicinals""[All Fields] OR ""Medicine""[MeSH Terms] OR ""Medicine""[All Fields] OR ""medicine s""[All Fields] OR ""Medicines""[All Fields]) AND ""east asia""[Title/Abstract]) OR ((""medicin""[All Fields] OR ""medicinal""[All Fields] OR ""medicinally""[All Fields] OR ""medicinals""[All Fields] OR ""Medicine""[MeSH Terms] OR ""Medicine""[All Fields] OR ""medicine s""[All Fields] OR ""Medicines""[All Fields]) AND ""far east""[Title/Abstract]) OR (""East""[All Fields] AND ""medicine far""[Title/Abstract]) OR ((""East""[All Fields] AND (""medicin""[All Fields] OR ""medicinal""[All Fields] OR ""medicinally""[All Fields] OR ""medicinals""[All Fields] OR ""Medicine""[MeSH Terms] OR ""Medicine""[All Fields] OR ""medicine s""[All Fields] OR ""Medicines""[All Fields])) AND ""Far""[Title/Abstract]) OR ""far east medicine""[Title/Abstract] OR ""far east medicines""[Title/Abstract] OR ((""medicin""[All Fields] OR ""medicinal""[All Fields] OR ""medicinally""[All Fields] OR ""medicinals""[All Fields] OR ""Medicine""[MeSH Terms] OR ""Medicine""[All Fields] OR ""medicine s""[All Fields] OR ""Medicines""[All Fields]) AND ""far east""[Title/Abstract]))) OR (""medicine, chinese traditional""[MeSH Terms] OR (""zhong yi xue""[Title/Abstract] OR ((chung i[Author] OR chung i[Investigator]) AND ""Hsueh""[Title/Abstract]) OR (""Hsueh""[All Fields] AND ""chung i""[Title/Abstract]) OR ""traditional medicine chinese""[Title/Abstract] OR ""chinese traditional medicine""[Title/Abstract] OR ""traditional chinese medicine""[Title/Abstract] OR ""chinese medicine traditional""[Title/Abstract] OR ""traditional tongue diagnosis""[Title/Abstract] OR (((""Tongue""[MeSH Terms] OR ""Tongue""[All Fields] OR ""tongues""[All Fields] OR ""tongue s""[All Fields]) AND (""diagnosable""[All Fields] OR ""diagnosi""[All Fields] OR ""Diagnosis""[MeSH Terms] OR ""Diagnosis""[All Fields] OR ""diagnose""[All Fields] OR ""diagnosed""[All Fields] OR ""Diagnoses""[All Fields] OR ""diagnosing""[All Fields] OR ""Diagnosis""[MeSH Subheading])) AND ""Traditional""[Title/Abstract]) OR (((""Tongue""[MeSH Terms] OR ""Tongue""[All Fields] OR ""tongues""[All Fields] OR ""tongue s""[All Fields]) AND (""diagnosable""[All Fields] OR ""diagnosi""[All Fields] OR ""Diagnosis""[MeSH Terms] OR ""Diagnosis""[All Fields] OR ""diagnose""[All Fields] OR ""diagnosed""[All Fields] OR ""Diagnoses""[All Fields] OR ""diagnosing""[All Fields] OR ""Diagnosis""[MeSH Subheading])) AND ""Traditional""[Title/Abstract]) OR ((""tradition""[All Fields] OR ""tradition s""[All Fields] OR ""Traditional""[All Fields] OR ""traditionals""[All Fields] OR ""traditions""[All Fields]) AND ""tongue diagnoses""[Title/Abstract]) OR ((""tradition""[All Fields] OR ""tradition s""[All Fields] OR ""Traditional""[All Fields] OR ""traditionals""[All Fields] OR ""traditions""[All Fields]) AND ""tongue assessment""[Title/Abstract]) OR ((""Tongue""[MeSH Terms] OR ""Tongue""[All Fields] OR ""tongues""[All Fields] OR ""tongue s""[All Fields]) AND ""assessment traditional""[Title/Abstract]) OR ((""tradition""[All Fields] OR ""tradition s""[All Fields] OR ""Traditional""[All Fields] OR ""traditionals""[All Fields] OR ""traditions""[All Fields]) AND ""tongue assessments""[Title/Abstract]))) OR ""Electroacupuncture""[MeSH Terms]","106,988",09:58:49

32,"(""Medicine, Chinese Traditional""[Mesh]) OR ((((((((((((((Zhong Yi Xue[Title/Abstract]) OR (Chung I Hsueh[Title/Abstract])) OR (Hsueh, Chung I[Title/Abstract])) OR (Traditional Medicine, Chinese[Title/Abstract])) OR (Chinese Traditional Medicine[Title/Abstract])) OR (Traditional Chinese Medicine[Title/Abstract])) OR (Chinese Medicine, Traditional[Title/Abstract])) OR (Traditional Tongue Diagnosis[Title/Abstract])) OR (Tongue Diagnoses, Traditional[Title/Abstract])) OR (Tongue Diagnosis, Traditional[Title/Abstract])) OR (Traditional Tongue Diagnoses[Title/Abstract])) OR (Traditional Tongue Assessment[Title/Abstract])) OR (Tongue Assessment, Traditional[Title/Abstract])) OR (Traditional Tongue Assessments[Title/Abstract]))",,,"""medicine, chinese traditional""[MeSH Terms] OR (""zhong yi xue""[Title/Abstract] OR ((chung i[Author] OR chung i[Investigator]) AND ""Hsueh""[Title/Abstract]) OR (""Hsueh""[All Fields] AND ""chung i""[Title/Abstract]) OR ""traditional medicine chinese""[Title/Abstract] OR ""chinese traditional medicine""[Title/Abstract] OR ""traditional chinese medicine""[Title/Abstract] OR ""chinese medicine traditional""[Title/Abstract] OR ""traditional tongue diagnosis""[Title/Abstract] OR (((""Tongue""[MeSH Terms] OR ""Tongue""[All Fields] OR ""tongues""[All Fields] OR ""tongue s""[All Fields]) AND (""diagnosable""[All Fields] OR ""diagnosi""[All Fields] OR ""Diagnosis""[MeSH Terms] OR ""Diagnosis""[All Fields] OR ""diagnose""[All Fields] OR ""diagnosed""[All Fields] OR ""Diagnoses""[All Fields] OR ""diagnosing""[All Fields] OR ""Diagnosis""[MeSH Subheading])) AND ""Traditional""[Title/Abstract]) OR (((""Tongue""[MeSH Terms] OR ""Tongue""[All Fields] OR ""tongues""[All Fields] OR ""tongue s""[All Fields]) AND (""diagnosable""[All Fields] OR ""diagnosi""[All Fields] OR ""Diagnosis""[MeSH Terms] OR ""Diagnosis""[All Fields] OR ""diagnose""[All Fields] OR ""diagnosed""[All Fields] OR ""Diagnoses""[All Fields] OR ""diagnosing""[All Fields] OR ""Diagnosis""[MeSH Subheading])) AND ""Traditional""[Title/Abstract]) OR ((""tradition""[All Fields] OR ""tradition s""[All Fields] OR ""Traditional""[All Fields] OR ""traditionals""[All Fields] OR ""traditions""[All Fields]) AND ""tongue diagnoses""[Title/Abstract]) OR ((""tradition""[All Fields] OR ""tradition s""[All Fields] OR ""Traditional""[All Fields] OR ""traditionals""[All Fields] OR ""traditions""[All Fields]) AND ""tongue assessment""[Title/Abstract]) OR ((""Tongue""[MeSH Terms] OR ""Tongue""[All Fields] OR ""tongues""[All Fields] OR ""tongue s""[All Fields]) AND ""assessment traditional""[Title/Abstract]) OR ((""tradition""[All Fields] OR ""tradition s""[All Fields] OR ""Traditional""[All Fields] OR ""traditionals""[All Fields] OR ""traditions""[All Fields]) AND ""tongue assessments""[Title/Abstract]))","56,244",09:57:49

31,"(""Medicine, East Asian Traditional""[Mesh]) OR ((((((((((((((((((((((((((((Oriental Traditional Medicine[Title/Abstract]) OR (Oriental Medicine, Traditional[Title/Abstract])) OR (Medicine, Traditional Oriental[Title/Abstract])) OR (Traditional Oriental Medicine[Title/Abstract])) OR (Traditional Oriental Medicines[Title/Abstract])) OR (Traditional Medicine, Oriental[Title/Abstract])) OR (Medicine, Oriental Traditional[Title/Abstract])) OR (Medicine, Traditional, East Asia[Title/Abstract])) OR (Traditional Medicine, East Asia[Title/Abstract])) OR (Traditional Far Eastern Medicine[Title/Abstract])) OR (East Asian Traditional Medicine[Title/Abstract])) OR (Traditional East Asian Medicine[Title/Abstract])) OR (East Asian Medicine[Title/Abstract])) OR (East Asian Medicines[Title/Abstract])) OR (Medicine, East Asian[Title/Abstract])) OR (Medicine, Oriental[Title/Abstract])) OR (Oriental Medicine[Title/Abstract])) OR (Medicine, East Asia[Title/Abstract])) OR (Asia Medicines, East[Title/Abstract])) OR (East Asia Medicine[Title/Abstract])) OR (East Asia Medicines[Title/Abstract])) OR (Medicines, East Asia[Title/Abstract])) OR (Medicine, Far East[Title/Abstract])) OR (East Medicine, Far[Title/Abstract])) OR (East Medicines, Far[Title/Abstract])) OR (Far East Medicine[Title/Abstract])) OR (Far East Medicines[Title/Abstract])) OR (Medicines, Far East[Title/Abstract]))",,,"""medicine, east asian traditional""[MeSH Terms] OR (""oriental traditional medicine""[Title/Abstract] OR ((""asian people""[MeSH Terms] OR (""Asian""[All Fields] AND ""people""[All Fields]) OR ""asian people""[All Fields] OR ""Oriental""[All Fields] OR ""orientals""[All Fields]) AND ""medicine traditional""[Title/Abstract]) OR ((""medicin""[All Fields] OR ""medicinal""[All Fields] OR ""medicinally""[All Fields] OR ""medicinals""[All Fields] OR ""Medicine""[MeSH Terms] OR ""Medicine""[All Fields] OR ""medicine s""[All Fields] OR ""Medicines""[All Fields]) AND ""traditional oriental""[Title/Abstract]) OR ""traditional oriental medicine""[Title/Abstract] OR ""traditional oriental medicines""[Title/Abstract] OR ""traditional medicine oriental""[Title/Abstract] OR ((""medicin""[All Fields] OR ""medicinal""[All Fields] OR ""medicinally""[All Fields] OR ""medicinals""[All Fields] OR ""Medicine""[MeSH Terms] OR ""Medicine""[All Fields] OR ""medicine s""[All Fields] OR ""Medicines""[All Fields]) AND ""oriental traditional""[Title/Abstract]) OR ((""medicine, traditional""[MeSH Terms] OR (""Medicine""[All Fields] AND ""Traditional""[All Fields]) OR ""traditional medicine""[All Fields] OR ""medicine traditional""[All Fields]) AND ""east asia""[Title/Abstract]) OR ((""tradition""[All Fields] OR ""tradition s""[All Fields] OR ""Traditional""[All Fields] OR ""traditionals""[All Fields] OR ""traditions""[All Fields]) AND ""medicine east asia""[Title/Abstract]) OR ((""tradition""[All Fields] OR ""tradition s""[All Fields] OR ""Traditional""[All Fields] OR ""traditionals""[All Fields] OR ""traditions""[All Fields]) AND ""far eastern medicine""[Title/Abstract]) OR ""east asian traditional medicine""[Title/Abstract] OR ""traditional east asian medicine""[Title/Abstract] OR ""east asian medicine""[Title/Abstract] OR ""east asian medicines""[Title/Abstract] OR ""medicine east asian""[Title/Abstract] OR ""medicine oriental""[Title/Abstract] OR ""oriental medicine""[Title/Abstract] OR ""medicine east asia""[Title/Abstract] OR (((""Asia""[MeSH Terms] OR ""Asia""[All Fields]) AND (""medicin""[All Fields] OR ""medicinal""[All Fields] OR ""medicinally""[All Fields] OR ""medicinals""[All Fields] OR ""Medicine""[MeSH Terms] OR ""Medicine""[All Fields] OR ""medicine s""[All Fields] OR ""Medicines""[All Fields])) AND ""East""[Title/Abstract]) OR ""east asia medicine""[Title/Abstract] OR (""East""[All Fields] AND ""asia medicines""[Title/Abstract]) OR ((""medicin""[All Fields] OR ""medicinal""[All Fields] OR ""medicinally""[All Fields] OR ""medicinals""[All Fields] OR ""Medicine""[MeSH Terms] OR ""Medicine""[All Fields] OR ""medicine s""[All Fields] OR ""Medicines""[All Fields]) AND ""east asia""[Title/Abstract]) OR ((""medicin""[All Fields] OR ""medicinal""[All Fields] OR ""medicinally""[All Fields] OR ""medicinals""[All Fields] OR ""Medicine""[MeSH Terms] OR ""Medicine""[All Fields] OR ""medicine s""[All Fields] OR ""Medicines""[All Fields]) AND ""far east""[Title/Abstract]) OR (""East""[All Fields] AND ""medicine far""[Title/Abstract]) OR ((""East""[All Fields] AND (""medicin""[All Fields] OR ""medicinal""[All Fields] OR ""medicinally""[All Fields] OR ""medicinals""[All Fields] OR ""Medicine""[MeSH Terms] OR ""Medicine""[All Fields] OR ""medicine s""[All Fields] OR ""Medicines""[All Fields])) AND ""Far""[Title/Abstract]) OR ""far east medicine""[Title/Abstract] OR ""far east medicines""[Title/Abstract] OR ((""medicin""[All Fields] OR ""medicinal""[All Fields] OR ""medicinally""[All Fields] OR ""medicinals""[All Fields] OR ""Medicine""[MeSH Terms] OR ""Medicine""[All Fields] OR ""medicine s""[All Fields] OR ""Medicines""[All Fields]) AND ""far east""[Title/Abstract]))","48,685",09:57:23

30,"(""Moxibustion""[Mesh]) OR (Moxabustion[Title/Abstract])",,,"""Moxibustion""[MeSH Terms] OR ""Moxabustion""[Title/Abstract]","3,067",09:56:42

29,"(""Auriculotherapy""[Mesh]) OR (Auriculotherapies[Title/Abstract])",,,"""Auriculotherapy""[MeSH Terms] OR ""Auriculotherapies""[Title/Abstract]",665,09:56:05

28,"(""Acupuncture Therapy""[Mesh]) OR ((((((((((Acupuncture Treatment[Title/Abstract]) OR (Acupuncture Treatments[Title/Abstract])) OR (Treatment, Acupuncture[Title/Abstract])) OR (Therapy, Acupuncture[Title/Abstract])) OR (Pharmacoacupuncture Treatment[Title/Abstract])) OR (Treatment, Pharmacoacupuncture[Title/Abstract])) OR (Pharmacoacupuncture Therapy[Title/Abstract])) OR (Therapy, Pharmacoacupuncture[Title/Abstract])) OR (Acupotomy[Title/Abstract])) OR (Acupotomies[Title/Abstract]))",,,"""Acupuncture Therapy""[MeSH Terms] OR (""acupuncture treatment""[Title/Abstract] OR ""acupuncture treatments""[Title/Abstract] OR ""treatment acupuncture""[Title/Abstract] OR ""therapy acupuncture""[Title/Abstract] OR ""pharmacoacupuncture treatment""[Title/Abstract] OR ((""therapeutics""[MeSH Terms] OR ""therapeutics""[All Fields] OR ""Treatments""[All Fields] OR ""Therapy""[MeSH Subheading] OR ""Therapy""[All Fields] OR ""Treatment""[All Fields] OR ""treatment s""[All Fields]) AND ""Pharmacoacupuncture""[Title/Abstract]) OR ""pharmacoacupuncture therapy""[Title/Abstract] OR ((""therapeutics""[MeSH Terms] OR ""therapeutics""[All Fields] OR ""therapies""[All Fields] OR ""Therapy""[MeSH Subheading] OR ""Therapy""[All Fields] OR ""therapy s""[All Fields] OR ""therapys""[All Fields]) AND ""Pharmacoacupuncture""[Title/Abstract]) OR ""Acupotomy""[Title/Abstract] OR ""Acupotomies""[Title/Abstract])","33,008",09:55:30

27,"(""Acupuncture""[Mesh]) OR (Pharmacopuncture[Title/Abstract])",,,"""Acupuncture""[MeSH Terms] OR ""Pharmacopuncture""[Title/Abstract]","2,393",09:54:44

26,"(((((((((((((Zhong Yi Xue[Title/Abstract]) OR (Chung I Hsueh[Title/Abstract])) OR (Hsueh, Chung I[Title/Abstract])) OR (Traditional Medicine, Chinese[Title/Abstract])) OR (Chinese Traditional Medicine[Title/Abstract])) OR (Traditional Chinese Medicine[Title/Abstract])) OR (Chinese Medicine, Traditional[Title/Abstract])) OR (Traditional Tongue Diagnosis[Title/Abstract])) OR (Tongue Diagnoses, Traditional[Title/Abstract])) OR (Tongue Diagnosis, Traditional[Title/Abstract])) OR (Traditional Tongue Diagnoses[Title/Abstract])) OR (Traditional Tongue Assessment[Title/Abstract])) OR (Tongue Assessment, Traditional[Title/Abstract])) OR (Traditional Tongue Assessments[Title/Abstract])",,,"""zhong yi xue""[Title/Abstract] OR ((chung i[Author] OR chung i[Investigator]) AND ""Hsueh""[Title/Abstract]) OR (""Hsueh""[All Fields] AND ""chung i""[Title/Abstract]) OR ""traditional medicine chinese""[Title/Abstract] OR ""chinese traditional medicine""[Title/Abstract] OR ""traditional chinese medicine""[Title/Abstract] OR ""chinese medicine traditional""[Title/Abstract] OR ""traditional tongue diagnosis""[Title/Abstract] OR (((""Tongue""[MeSH Terms] OR ""Tongue""[All Fields] OR ""tongues""[All Fields] OR ""tongue s""[All Fields]) AND (""diagnosable""[All Fields] OR ""diagnosi""[All Fields] OR ""Diagnosis""[MeSH Terms] OR ""Diagnosis""[All Fields] OR ""diagnose""[All Fields] OR ""diagnosed""[All Fields] OR ""Diagnoses""[All Fields] OR ""diagnosing""[All Fields] OR ""Diagnosis""[MeSH Subheading])) AND ""Traditional""[Title/Abstract]) OR (((""Tongue""[MeSH Terms] OR ""Tongue""[All Fields] OR ""tongues""[All Fields] OR ""tongue s""[All Fields]) AND (""diagnosable""[All Fields] OR ""diagnosi""[All Fields] OR ""Diagnosis""[MeSH Terms] OR ""Diagnosis""[All Fields] OR ""diagnose""[All Fields] OR ""diagnosed""[All Fields] OR ""Diagnoses""[All Fields] OR ""diagnosing""[All Fields] OR ""Diagnosis""[MeSH Subheading])) AND ""Traditional""[Title/Abstract]) OR ((""tradition""[All Fields] OR ""tradition s""[All Fields] OR ""Traditional""[All Fields] OR ""traditionals""[All Fields] OR ""traditions""[All Fields]) AND ""tongue diagnoses""[Title/Abstract]) OR ((""tradition""[All Fields] OR ""tradition s""[All Fields] OR ""Traditional""[All Fields] OR ""traditionals""[All Fields] OR ""traditions""[All Fields]) AND ""tongue assessment""[Title/Abstract]) OR ((""Tongue""[MeSH Terms] OR ""Tongue""[All Fields] OR ""tongues""[All Fields] OR ""tongue s""[All Fields]) AND ""assessment traditional""[Title/Abstract]) OR ((""tradition""[All Fields] OR ""tradition s""[All Fields] OR ""Traditional""[All Fields] OR ""traditionals""[All Fields] OR ""traditions""[All Fields]) AND ""tongue assessments""[Title/Abstract])","42,042",09:50:34

25,"""Medicine, Chinese Traditional""[Mesh]",Most Recent,,"""medicine, chinese traditional""[MeSH Terms]","26,589",09:47:03

24,"(((((((((((((((((((((((((((Oriental Traditional Medicine[Title/Abstract]) OR (Oriental Medicine, Traditional[Title/Abstract])) OR (Medicine, Traditional Oriental[Title/Abstract])) OR (Traditional Oriental Medicine[Title/Abstract])) OR (Traditional Oriental Medicines[Title/Abstract])) OR (Traditional Medicine, Oriental[Title/Abstract])) OR (Medicine, Oriental Traditional[Title/Abstract])) OR (Medicine, Traditional, East Asia[Title/Abstract])) OR (Traditional Medicine, East Asia[Title/Abstract])) OR (Traditional Far Eastern Medicine[Title/Abstract])) OR (East Asian Traditional Medicine[Title/Abstract])) OR (Traditional East Asian Medicine[Title/Abstract])) OR (East Asian Medicine[Title/Abstract])) OR (East Asian Medicines[Title/Abstract])) OR (Medicine, East Asian[Title/Abstract])) OR (Medicine, Oriental[Title/Abstract])) OR (Oriental Medicine[Title/Abstract])) OR (Medicine, East Asia[Title/Abstract])) OR (Asia Medicines, East[Title/Abstract])) OR (East Asia Medicine[Title/Abstract])) OR (East Asia Medicines[Title/Abstract])) OR (Medicines, East Asia[Title/Abstract])) OR (Medicine, Far East[Title/Abstract])) OR (East Medicine, Far[Title/Abstract])) OR (East Medicines, Far[Title/Abstract])) OR (Far East Medicine[Title/Abstract])) OR (Far East Medicines[Title/Abstract])) OR (Medicines, Far East[Title/Abstract])",,,"""oriental traditional medicine""[Title/Abstract] OR ((""asian people""[MeSH Terms] OR (""Asian""[All Fields] AND ""people""[All Fields]) OR ""asian people""[All Fields] OR ""Oriental""[All Fields] OR ""orientals""[All Fields]) AND ""medicine traditional""[Title/Abstract]) OR ((""medicin""[All Fields] OR ""medicinal""[All Fields] OR ""medicinally""[All Fields] OR ""medicinals""[All Fields] OR ""Medicine""[MeSH Terms] OR ""Medicine""[All Fields] OR ""medicine s""[All Fields] OR ""Medicines""[All Fields]) AND ""traditional oriental""[Title/Abstract]) OR ""traditional oriental medicine""[Title/Abstract] OR ""traditional oriental medicines""[Title/Abstract] OR ""traditional medicine oriental""[Title/Abstract] OR ((""medicin""[All Fields] OR ""medicinal""[All Fields] OR ""medicinally""[All Fields] OR ""medicinals""[All Fields] OR ""Medicine""[MeSH Terms] OR ""Medicine""[All Fields] OR ""medicine s""[All Fields] OR ""Medicines""[All Fields]) AND ""oriental traditional""[Title/Abstract]) OR ((""medicine, traditional""[MeSH Terms] OR (""Medicine""[All Fields] AND ""Traditional""[All Fields]) OR ""traditional medicine""[All Fields] OR ""medicine traditional""[All Fields]) AND ""east asia""[Title/Abstract]) OR ((""tradition""[All Fields] OR ""tradition s""[All Fields] OR ""Traditional""[All Fields] OR ""traditionals""[All Fields] OR ""traditions""[All Fields]) AND ""medicine east asia""[Title/Abstract]) OR ((""tradition""[All Fields] OR ""tradition s""[All Fields] OR ""Traditional""[All Fields] OR ""traditionals""[All Fields] OR ""traditions""[All Fields]) AND ""far eastern medicine""[Title/Abstract]) OR ""east asian traditional medicine""[Title/Abstract] OR ""traditional east asian medicine""[Title/Abstract] OR ""east asian medicine""[Title/Abstract] OR ""east asian medicines""[Title/Abstract] OR ""medicine east asian""[Title/Abstract] OR ""medicine oriental""[Title/Abstract] OR ""oriental medicine""[Title/Abstract] OR ""medicine east asia""[Title/Abstract] OR (((""Asia""[MeSH Terms] OR ""Asia""[All Fields]) AND (""medicin""[All Fields] OR ""medicinal""[All Fields] OR ""medicinally""[All Fields] OR ""medicinals""[All Fields] OR ""Medicine""[MeSH Terms] OR ""Medicine""[All Fields] OR ""medicine s""[All Fields] OR ""Medicines""[All Fields])) AND ""East""[Title/Abstract]) OR ""east asia medicine""[Title/Abstract] OR (""East""[All Fields] AND ""asia medicines""[Title/Abstract]) OR ((""medicin""[All Fields] OR ""medicinal""[All Fields] OR ""medicinally""[All Fields] OR ""medicinals""[All Fields] OR ""Medicine""[MeSH Terms] OR ""Medicine""[All Fields] OR ""medicine s""[All Fields] OR ""Medicines""[All Fields]) AND ""east asia""[Title/Abstract]) OR ((""medicin""[All Fields] OR ""medicinal""[All Fields] OR ""medicinally""[All Fields] OR ""medicinals""[All Fields] OR ""Medicine""[MeSH Terms] OR ""Medicine""[All Fields] OR ""medicine s""[All Fields] OR ""Medicines""[All Fields]) AND ""far east""[Title/Abstract]) OR (""East""[All Fields] AND ""medicine far""[Title/Abstract]) OR ((""East""[All Fields] AND (""medicin""[All Fields] OR ""medicinal""[All Fields] OR ""medicinally""[All Fields] OR ""medicinals""[All Fields] OR ""Medicine""[MeSH Terms] OR ""Medicine""[All Fields] OR ""medicine s""[All Fields] OR ""Medicines""[All Fields])) AND ""Far""[Title/Abstract]) OR ""far east medicine""[Title/Abstract] OR ""far east medicines""[Title/Abstract] OR ((""medicin""[All Fields] OR ""medicinal""[All Fields] OR ""medicinally""[All Fields] OR ""medicinals""[All Fields] OR ""Medicine""[MeSH Terms] OR ""Medicine""[All Fields] OR ""medicine s""[All Fields] OR ""Medicines""[All Fields]) AND ""far east""[Title/Abstract])","19,154",09:46:14

23,"""Medicine, East Asian Traditional""[Mesh]",Most Recent,,"""medicine, east asian traditional""[MeSH Terms]","30,197",09:42:10

22,Moxabustion[Title/Abstract],,,"""Moxabustion""[Title/Abstract]",1,09:41:30

21,"""Moxibustion""[Mesh]",Most Recent,,"""Moxibustion""[MeSH Terms]","3,066",09:41:07

20,"""Electroacupuncture""[Mesh]",Most Recent,,"""Electroacupuncture""[MeSH Terms]","5,577",09:40:25

19,Auriculotherapies[Title/Abstract],,,"""Auriculotherapies""[Title/Abstract]",1,09:39:55

18,"""Auriculotherapy""[Mesh]",Most Recent,,"""Auriculotherapy""[MeSH Terms]",665,09:39:37

17,"(((((((((Acupuncture Treatment[Title/Abstract]) OR (Acupuncture Treatments[Title/Abstract])) OR (Treatment, Acupuncture[Title/Abstract])) OR (Therapy, Acupuncture[Title/Abstract])) OR (Pharmacoacupuncture Treatment[Title/Abstract])) OR (Treatment, Pharmacoacupuncture[Title/Abstract])) OR (Pharmacoacupuncture Therapy[Title/Abstract])) OR (Therapy, Pharmacoacupuncture[Title/Abstract])) OR (Acupotomy[Title/Abstract])) OR (Acupotomies[Title/Abstract])",,,"""acupuncture treatment""[Title/Abstract] OR ""acupuncture treatments""[Title/Abstract] OR ""treatment acupuncture""[Title/Abstract] OR ""therapy acupuncture""[Title/Abstract] OR ""pharmacoacupuncture treatment""[Title/Abstract] OR ((""therapeutics""[MeSH Terms] OR ""therapeutics""[All Fields] OR ""Treatments""[All Fields] OR ""Therapy""[MeSH Subheading] OR ""Therapy""[All Fields] OR ""Treatment""[All Fields] OR ""treatment s""[All Fields]) AND ""Pharmacoacupuncture""[Title/Abstract]) OR ""pharmacoacupuncture therapy""[Title/Abstract] OR ((""therapeutics""[MeSH Terms] OR ""therapeutics""[All Fields] OR ""therapies""[All Fields] OR ""Therapy""[MeSH Subheading] OR ""Therapy""[All Fields] OR ""therapy s""[All Fields] OR ""therapys""[All Fields]) AND ""Pharmacoacupuncture""[Title/Abstract]) OR ""Acupotomy""[Title/Abstract] OR ""Acupotomies""[Title/Abstract]","4,641",09:38:35

16,"""Acupuncture Therapy""[Mesh]",Most Recent,,"""Acupuncture Therapy""[MeSH Terms]","31,752",09:37:17

15,Pharmacopuncture[Title/Abstract],,,"""Pharmacopuncture""[Title/Abstract]",305,09:36:35

14,"""Acupuncture""[Mesh]",Most Recent,,"""Acupuncture""[MeSH Terms]","2,111",09:35:59

13,"((((""Embryo Transfer""[Mesh]) OR ((((((Embryo Transfers[Title/Abstract]) OR (Transfer, Embryo[Title/Abstract])) OR (Transfers, Embryo[Title/Abstract])) OR (Tubal Embryo Transfer[Title/Abstract])) OR (Tubal Embryo Stage Transfer[Title/Abstract])) OR (Blastocyst Transfer[Title/Abstract]))) OR ((""Reproductive Techniques, Assisted""[Mesh]) OR (((((((((((((((((Assisted Reproductive Technique[Title/Abstract]) OR (Reproductive Technique, Assisted[Title/Abstract])) OR (Technique, Assisted Reproductive[Title/Abstract])) OR (Techniques, Assisted Reproductive[Title/Abstract])) OR (Assisted Reproductive Technics[Title/Abstract])) OR (Assisted Reproductive Technic[Title/Abstract])) OR (Reproductive Technic, Assisted[Title/Abstract])) OR (Reproductive Technics, Assisted[Title/Abstract])) OR (Technic, Assisted Reproductive[Title/Abstract])) OR (Technics, Assisted Reproductive[Title/Abstract])) OR (Assisted Reproductive Techniques[Title/Abstract])) OR (Reproductive Technology, Assisted[Title/Abstract])) OR (Assisted Reproductive Technologies[Title/Abstract])) OR (Assisted Reproductive Technology[Title/Abstract])) OR (Reproductive Technologies, Assisted[Title/Abstract])) OR (Technologies, Assisted Reproductive[Title/Abstract])) OR (Technology, Assisted Reproductive[Title/Abstract])))) OR ((""Fertilization in Vitro""[Mesh]) OR (((((((((((((In Vitro Fertilization[Title/Abstract]) OR (In Vitro Fertilizations[Title/Abstract])) OR (Test-Tube Fertilization[Title/Abstract])) OR (Fertilizations, Test-Tube[Title/Abstract])) OR (Fertilization, Test-Tube[Title/Abstract])) OR (Test Tube Fertilization[Title/Abstract])) OR (Test-Tube Fertilizations[Title/Abstract])) OR (Fertilizations in Vitro[Title/Abstract])) OR (Test-Tube Babies[Title/Abstract])) OR (Babies, Test-Tube[Title/Abstract])) OR (Baby, Test-Tube[Title/Abstract])) OR (Test Tube Babies[Title/Abstract])) OR (Test-Tube Baby[Title/Abstract])))) OR ((""Oocytes""[Mesh]) OR (((Oocyte[Title/Abstract]) OR (Ovocytes[Title/Abstract])) OR (Ovocyte[Title/Abstract])))",,,"""Embryo Transfer""[MeSH Terms] OR (""embryo transfers""[Title/Abstract] OR ""transfer embryo""[Title/Abstract] OR ""transfers embryo""[Title/Abstract] OR ""tubal embryo transfer""[Title/Abstract] OR ""tubal embryo stage transfer""[Title/Abstract] OR ""blastocyst transfer""[Title/Abstract]) OR (""reproductive techniques, assisted""[MeSH Terms] OR (""assisted reproductive technique""[Title/Abstract] OR ""reproductive technique assisted""[Title/Abstract] OR ((""methods""[MeSH Terms] OR ""methods""[All Fields] OR ""Technique""[All Fields] OR ""methods""[MeSH Subheading] OR ""Techniques""[All Fields] OR ""technique s""[All Fields]) AND ""assisted reproductive""[Title/Abstract]) OR ((""methods""[MeSH Terms] OR ""methods""[All Fields] OR ""Technique""[All Fields] OR ""methods""[MeSH Subheading] OR ""Techniques""[All Fields] OR ""technique s""[All Fields]) AND ""assisted reproductive""[Title/Abstract]) OR ""assisted reproductive technics""[Title/Abstract] OR ""assisted reproductive technic""[Title/Abstract] OR (((""reproduction""[MeSH Terms] OR ""reproduction""[All Fields] OR ""reproductions""[All Fields] OR ""Reproductive""[All Fields] OR ""reproductively""[All Fields] OR ""reproductives""[All Fields] OR ""reproductivity""[All Fields]) AND (""Technic""[All Fields] OR ""Technics""[All Fields])) AND ""Assisted""[Title/Abstract]) OR (((""reproduction""[MeSH Terms] OR ""reproduction""[All Fields] OR ""reproductions""[All Fields] OR ""Reproductive""[All Fields] OR ""reproductively""[All Fields] OR ""reproductives""[All Fields] OR ""reproductivity""[All Fields]) AND (""Technic""[All Fields] OR ""Technics""[All Fields])) AND ""Assisted""[Title/Abstract]) OR ((""Technic""[All Fields] OR ""Technics""[All Fields]) AND ""assisted reproductive""[Title/Abstract]) OR ((""Technic""[All Fields] OR ""Technics""[All Fields]) AND ""assisted reproductive""[Title/Abstract]) OR ""assisted reproductive techniques""[Title/Abstract] OR ((""reproduction""[MeSH Terms] OR ""reproduction""[All Fields] OR ""reproductions""[All Fields] OR ""Reproductive""[All Fields] OR ""reproductively""[All Fields] OR ""reproductives""[All Fields] OR ""reproductivity""[All Fields]) AND ""technology assisted""[Title/Abstract]) OR ""assisted reproductive technologies""[Title/Abstract] OR ""assisted reproductive technology""[Title/Abstract] OR ""reproductive technologies assisted""[Title/Abstract] OR ""technologies assisted reproductive""[Title/Abstract] OR ""technology assisted reproductive""[Title/Abstract])) OR (""Fertilization in Vitro""[MeSH Terms] OR (""in vitro fertilization""[Title/Abstract] OR ""in vitro fertilizations""[Title/Abstract] OR ""test tube fertilization""[Title/Abstract] OR ((""fertilisability""[All Fields] OR ""fertilisable""[All Fields] OR ""fertilisation""[All Fields] OR ""Fertilization""[MeSH Terms] OR ""Fertilization""[All Fields] OR ""fertilisations""[All Fields] OR ""fertilise""[All Fields] OR ""fertilised""[All Fields] OR ""fertilisers""[All Fields] OR ""fertilizers""[Pharmacological Action] OR ""fertilizers""[Supplementary Concept] OR ""fertilizers""[All Fields] OR ""fertilizers""[MeSH Terms] OR ""fertilises""[All Fields] OR ""fertilising""[All Fields] OR ""Fertilizations""[All Fields] OR ""fertilize""[All Fields] OR ""fertilized""[All Fields] OR ""fertiliser""[All Fields] OR ""fertilizer""[All Fields] OR ""fertilizes""[All Fields] OR ""fertilizing""[All Fields]) AND ""Test-Tube""[Title/Abstract]) OR ""fertilization test tube""[Title/Abstract] OR ""test tube fertilization""[Title/Abstract] OR (""Test-Tube""[All Fields] AND ""Fertilizations""[Title/Abstract]) OR ""fertilizations in vitro""[Title/Abstract] OR ""test tube babies""[Title/Abstract] OR ((""baby s""[All Fields] OR ""babys""[All Fields] OR ""infant""[MeSH Terms] OR ""infant""[All Fields] OR ""Babies""[All Fields]) AND ""Test-Tube""[Title/Abstract]) OR ((""infant, newborn""[MeSH Terms] OR (""infant""[All Fields] AND ""newborn""[All Fields]) OR ""newborn infant""[All Fields] OR ""Baby""[All Fields] OR ""infant""[MeSH Terms] OR ""infant""[All Fields]) AND ""Test-Tube""[Title/Abstract]) OR ""test tube babies""[Title/Abstract] OR ""test tube baby""[Title/Abstract])) OR (""Oocytes""[MeSH Terms] OR (""Oocyte""[Title/Abstract] OR ""Ovocytes""[Title/Abstract] OR ""Ovocyte""[Title/Abstract]))","152,515",09:34:55

12,"(""Oocytes""[Mesh]) OR (((Oocyte[Title/Abstract]) OR (Ovocytes[Title/Abstract])) OR (Ovocyte[Title/Abstract]))",,,"""Oocytes""[MeSH Terms] OR ""Oocyte""[Title/Abstract] OR ""Ovocytes""[Title/Abstract] OR ""Ovocyte""[Title/Abstract]","75,956",09:34:23

11,"(""Fertilization in Vitro""[Mesh]) OR (((((((((((((In Vitro Fertilization[Title/Abstract]) OR (In Vitro Fertilizations[Title/Abstract])) OR (Test-Tube Fertilization[Title/Abstract])) OR (Fertilizations, Test-Tube[Title/Abstract])) OR (Fertilization, Test-Tube[Title/Abstract])) OR (Test Tube Fertilization[Title/Abstract])) OR (Test-Tube Fertilizations[Title/Abstract])) OR (Fertilizations in Vitro[Title/Abstract])) OR (Test-Tube Babies[Title/Abstract])) OR (Babies, Test-Tube[Title/Abstract])) OR (Baby, Test-Tube[Title/Abstract])) OR (Test Tube Babies[Title/Abstract])) OR (Test-Tube Baby[Title/Abstract]))",,,"""Fertilization in Vitro""[MeSH Terms] OR (""in vitro fertilization""[Title/Abstract] OR ""in vitro fertilizations""[Title/Abstract] OR ""test tube fertilization""[Title/Abstract] OR ((""fertilisability""[All Fields] OR ""fertilisable""[All Fields] OR ""fertilisation""[All Fields] OR ""Fertilization""[MeSH Terms] OR ""Fertilization""[All Fields] OR ""fertilisations""[All Fields] OR ""fertilise""[All Fields] OR ""fertilised""[All Fields] OR ""fertilisers""[All Fields] OR ""fertilizers""[Pharmacological Action] OR ""fertilizers""[Supplementary Concept] OR ""fertilizers""[All Fields] OR ""fertilizers""[MeSH Terms] OR ""fertilises""[All Fields] OR ""fertilising""[All Fields] OR ""Fertilizations""[All Fields] OR ""fertilize""[All Fields] OR ""fertilized""[All Fields] OR ""fertiliser""[All Fields] OR ""fertilizer""[All Fields] OR ""fertilizes""[All Fields] OR ""fertilizing""[All Fields]) AND ""Test-Tube""[Title/Abstract]) OR ""fertilization test tube""[Title/Abstract] OR ""test tube fertilization""[Title/Abstract] OR (""Test-Tube""[All Fields] AND ""Fertilizations""[Title/Abstract]) OR ""fertilizations in vitro""[Title/Abstract] OR ""test tube babies""[Title/Abstract] OR ((""baby s""[All Fields] OR ""babys""[All Fields] OR ""infant""[MeSH Terms] OR ""infant""[All Fields] OR ""Babies""[All Fields]) AND ""Test-Tube""[Title/Abstract]) OR ((""infant, newborn""[MeSH Terms] OR (""infant""[All Fields] AND ""newborn""[All Fields]) OR ""newborn infant""[All Fields] OR ""Baby""[All Fields] OR ""infant""[MeSH Terms] OR ""infant""[All Fields]) AND ""Test-Tube""[Title/Abstract]) OR ""test tube babies""[Title/Abstract] OR ""test tube baby""[Title/Abstract])","54,316",09:33:15

10,"(""Reproductive Techniques, Assisted""[Mesh]) OR (((((((((((((((((Assisted Reproductive Technique[Title/Abstract]) OR (Reproductive Technique, Assisted[Title/Abstract])) OR (Technique, Assisted Reproductive[Title/Abstract])) OR (Techniques, Assisted Reproductive[Title/Abstract])) OR (Assisted Reproductive Technics[Title/Abstract])) OR (Assisted Reproductive Technic[Title/Abstract])) OR (Reproductive Technic, Assisted[Title/Abstract])) OR (Reproductive Technics, Assisted[Title/Abstract])) OR (Technic, Assisted Reproductive[Title/Abstract])) OR (Technics, Assisted Reproductive[Title/Abstract])) OR (Assisted Reproductive Techniques[Title/Abstract])) OR (Reproductive Technology, Assisted[Title/Abstract])) OR (Assisted Reproductive Technologies[Title/Abstract])) OR (Assisted Reproductive Technology[Title/Abstract])) OR (Reproductive Technologies, Assisted[Title/Abstract])) OR (Technologies, Assisted Reproductive[Title/Abstract])) OR (Technology, Assisted Reproductive[Title/Abstract]))",,,"""reproductive techniques, assisted""[MeSH Terms] OR (""assisted reproductive technique""[Title/Abstract] OR ""reproductive technique assisted""[Title/Abstract] OR ((""methods""[MeSH Terms] OR ""methods""[All Fields] OR ""Technique""[All Fields] OR ""methods""[MeSH Subheading] OR ""Techniques""[All Fields] OR ""technique s""[All Fields]) AND ""assisted reproductive""[Title/Abstract]) OR ((""methods""[MeSH Terms] OR ""methods""[All Fields] OR ""Technique""[All Fields] OR ""methods""[MeSH Subheading] OR ""Techniques""[All Fields] OR ""technique s""[All Fields]) AND ""assisted reproductive""[Title/Abstract]) OR ""assisted reproductive technics""[Title/Abstract] OR ""assisted reproductive technic""[Title/Abstract] OR (((""reproduction""[MeSH Terms] OR ""reproduction""[All Fields] OR ""reproductions""[All Fields] OR ""Reproductive""[All Fields] OR ""reproductively""[All Fields] OR ""reproductives""[All Fields] OR ""reproductivity""[All Fields]) AND (""Technic""[All Fields] OR ""Technics""[All Fields])) AND ""Assisted""[Title/Abstract]) OR (((""reproduction""[MeSH Terms] OR ""reproduction""[All Fields] OR ""reproductions""[All Fields] OR ""Reproductive""[All Fields] OR ""reproductively""[All Fields] OR ""reproductives""[All Fields] OR ""reproductivity""[All Fields]) AND (""Technic""[All Fields] OR ""Technics""[All Fields])) AND ""Assisted""[Title/Abstract]) OR ((""Technic""[All Fields] OR ""Technics""[All Fields]) AND ""assisted reproductive""[Title/Abstract]) OR ((""Technic""[All Fields] OR ""Technics""[All Fields]) AND ""assisted reproductive""[Title/Abstract]) OR ""assisted reproductive techniques""[Title/Abstract] OR ((""reproduction""[MeSH Terms] OR ""reproduction""[All Fields] OR ""reproductions""[All Fields] OR ""Reproductive""[All Fields] OR ""reproductively""[All Fields] OR ""reproductives""[All Fields] OR ""reproductivity""[All Fields]) AND ""technology assisted""[Title/Abstract]) OR ""assisted reproductive technologies""[Title/Abstract] OR ""assisted reproductive technology""[Title/Abstract] OR ""reproductive technologies assisted""[Title/Abstract] OR ""technologies assisted reproductive""[Title/Abstract] OR ""technology assisted reproductive""[Title/Abstract])","91,913",09:29:07

9,"(""Embryo Transfer""[Mesh]) OR ((((((Embryo Transfers[Title/Abstract]) OR (Transfer, Embryo[Title/Abstract])) OR (Transfers, Embryo[Title/Abstract])) OR (Tubal Embryo Transfer[Title/Abstract])) OR (Tubal Embryo Stage Transfer[Title/Abstract])) OR (Blastocyst Transfer[Title/Abstract]))",,,"""Embryo Transfer""[MeSH Terms] OR ""embryo transfers""[Title/Abstract] OR ""transfer embryo""[Title/Abstract] OR ""transfers embryo""[Title/Abstract] OR ""tubal embryo transfer""[Title/Abstract] OR ""tubal embryo stage transfer""[Title/Abstract] OR ""blastocyst transfer""[Title/Abstract]","20,883",09:28:37

8,((Oocyte[Title/Abstract]) OR (Ovocytes[Title/Abstract])) OR (Ovocyte[Title/Abstract]),,,"""Oocyte""[Title/Abstract] OR ""Ovocytes""[Title/Abstract] OR ""Ovocyte""[Title/Abstract]","49,273",09:27:53

7,"""Oocytes""[Mesh]",Most Recent,,"""Oocytes""[MeSH Terms]","52,911",09:26:49

6,"((((((((((((In Vitro Fertilization[Title/Abstract]) OR (In Vitro Fertilizations[Title/Abstract])) OR (Test-Tube Fertilization[Title/Abstract])) OR (Fertilizations, Test-Tube[Title/Abstract])) OR (Fertilization, Test-Tube[Title/Abstract])) OR (Test Tube Fertilization[Title/Abstract])) OR (Test-Tube Fertilizations[Title/Abstract])) OR (Fertilizations in Vitro[Title/Abstract])) OR (Test-Tube Babies[Title/Abstract])) OR (Babies, Test-Tube[Title/Abstract])) OR (Baby, Test-Tube[Title/Abstract])) OR (Test Tube Babies[Title/Abstract])) OR (Test-Tube Baby[Title/Abstract])",,,"""in vitro fertilization""[Title/Abstract] OR ""in vitro fertilizations""[Title/Abstract] OR ""test tube fertilization""[Title/Abstract] OR ((""fertilisability""[All Fields] OR ""fertilisable""[All Fields] OR ""fertilisation""[All Fields] OR ""Fertilization""[MeSH Terms] OR ""Fertilization""[All Fields] OR ""fertilisations""[All Fields] OR ""fertilise""[All Fields] OR ""fertilised""[All Fields] OR ""fertilisers""[All Fields] OR ""fertilizers""[Pharmacological Action] OR ""fertilizers""[Supplementary Concept] OR ""fertilizers""[All Fields] OR ""fertilizers""[MeSH Terms] OR ""fertilises""[All Fields] OR ""fertilising""[All Fields] OR ""Fertilizations""[All Fields] OR ""fertilize""[All Fields] OR ""fertilized""[All Fields] OR ""fertiliser""[All Fields] OR ""fertilizer""[All Fields] OR ""fertilizes""[All Fields] OR ""fertilizing""[All Fields]) AND ""Test-Tube""[Title/Abstract]) OR ""fertilization test tube""[Title/Abstract] OR ""test tube fertilization""[Title/Abstract] OR (""Test-Tube""[All Fields] AND ""Fertilizations""[Title/Abstract]) OR ""fertilizations in vitro""[Title/Abstract] OR ""test tube babies""[Title/Abstract] OR ((""baby s""[All Fields] OR ""babys""[All Fields] OR ""infant""[MeSH Terms] OR ""infant""[All Fields] OR ""Babies""[All Fields]) AND ""Test-Tube""[Title/Abstract]) OR ((""infant, newborn""[MeSH Terms] OR (""infant""[All Fields] AND ""newborn""[All Fields]) OR ""newborn infant""[All Fields] OR ""Baby""[All Fields] OR ""infant""[MeSH Terms] OR ""infant""[All Fields]) AND ""Test-Tube""[Title/Abstract]) OR ""test tube babies""[Title/Abstract] OR ""test tube baby""[Title/Abstract]","29,501",09:25:59

5,"""Fertilization in Vitro""[Mesh]",Most Recent,,"""Fertilization in Vitro""[MeSH Terms]","43,486",09:23:59

4,"((((((((((((((((Assisted Reproductive Technique[Title/Abstract]) OR (Reproductive Technique, Assisted[Title/Abstract])) OR (Technique, Assisted Reproductive[Title/Abstract])) OR (Techniques, Assisted Reproductive[Title/Abstract])) OR (Assisted Reproductive Technics[Title/Abstract])) OR (Assisted Reproductive Technic[Title/Abstract])) OR (Reproductive Technic, Assisted[Title/Abstract])) OR (Reproductive Technics, Assisted[Title/Abstract])) OR (Technic, Assisted Reproductive[Title/Abstract])) OR (Technics, Assisted Reproductive[Title/Abstract])) OR (Assisted Reproductive Techniques[Title/Abstract])) OR (Reproductive Technology, Assisted[Title/Abstract])) OR (Assisted Reproductive Technologies[Title/Abstract])) OR (Assisted Reproductive Technology[Title/Abstract])) OR (Reproductive Technologies, Assisted[Title/Abstract])) OR (Technologies, Assisted Reproductive[Title/Abstract])) OR (Technology, Assisted Reproductive[Title/Abstract])",,,"""assisted reproductive technique""[Title/Abstract] OR ""reproductive technique assisted""[Title/Abstract] OR ((""methods""[MeSH Terms] OR ""methods""[All Fields] OR ""Technique""[All Fields] OR ""methods""[MeSH Subheading] OR ""Techniques""[All Fields] OR ""technique s""[All Fields]) AND ""assisted reproductive""[Title/Abstract]) OR ((""methods""[MeSH Terms] OR ""methods""[All Fields] OR ""Technique""[All Fields] OR ""methods""[MeSH Subheading] OR ""Techniques""[All Fields] OR ""technique s""[All Fields]) AND ""assisted reproductive""[Title/Abstract]) OR ""assisted reproductive technics""[Title/Abstract] OR ""assisted reproductive technic""[Title/Abstract] OR (((""reproduction""[MeSH Terms] OR ""reproduction""[All Fields] OR ""reproductions""[All Fields] OR ""Reproductive""[All Fields] OR ""reproductively""[All Fields] OR ""reproductives""[All Fields] OR ""reproductivity""[All Fields]) AND (""Technic""[All Fields] OR ""Technics""[All Fields])) AND ""Assisted""[Title/Abstract]) OR (((""reproduction""[MeSH Terms] OR ""reproduction""[All Fields] OR ""reproductions""[All Fields] OR ""Reproductive""[All Fields] OR ""reproductively""[All Fields] OR ""reproductives""[All Fields] OR ""reproductivity""[All Fields]) AND (""Technic""[All Fields] OR ""Technics""[All Fields])) AND ""Assisted""[Title/Abstract]) OR ((""Technic""[All Fields] OR ""Technics""[All Fields]) AND ""assisted reproductive""[Title/Abstract]) OR ((""Technic""[All Fields] OR ""Technics""[All Fields]) AND ""assisted reproductive""[Title/Abstract]) OR ""assisted reproductive techniques""[Title/Abstract] OR ((""reproduction""[MeSH Terms] OR ""reproduction""[All Fields] OR ""reproductions""[All Fields] OR ""Reproductive""[All Fields] OR ""reproductively""[All Fields] OR ""reproductives""[All Fields] OR ""reproductivity""[All Fields]) AND ""technology assisted""[Title/Abstract]) OR ""assisted reproductive technologies""[Title/Abstract] OR ""assisted reproductive technology""[Title/Abstract] OR ""reproductive technologies assisted""[Title/Abstract] OR ""technologies assisted reproductive""[Title/Abstract] OR ""technology assisted reproductive""[Title/Abstract]","16,042",09:23:09

3,"""Reproductive Techniques, Assisted""[Mesh]",Most Recent,,"""reproductive techniques, assisted""[MeSH Terms]","85,294",09:20:24

2,"(((((Embryo Transfers[Title/Abstract]) OR (Transfer, Embryo[Title/Abstract])) OR (Transfers, Embryo[Title/Abstract])) OR (Tubal Embryo Transfer[Title/Abstract])) OR (Tubal Embryo Stage Transfer[Title/Abstract])) OR (Blastocyst Transfer[Title/Abstract])",,,"""embryo transfers""[Title/Abstract] OR ""transfer embryo""[Title/Abstract] OR ""transfers embryo""[Title/Abstract] OR ""tubal embryo transfer""[Title/Abstract] OR ""tubal embryo stage transfer""[Title/Abstract] OR ""blastocyst transfer""[Title/Abstract]","3,646",09:19:37

1,"""Embryo Transfer""[Mesh]",Most Recent,,"""Embryo Transfer""[MeSH Terms]","19,729",09:18:22
