# Supplementary material for: Acupuncture as an adjunctive therapy on embryo transfer day: a systematic review and meta-analysis of clinical pregnancy and live birth outcomes
Source: Front Reprod Health. 2025 Sep 23;7:1673144. doi: 10.3389/frph.2025.1673144 (PMC12500596; doi:10.3389/frph.2025.1673144)
Supplement: Supplementary file 2 [file Datasheet2.docx]

Search Name:

Date Run: 18/05/2025 00:33:03

Comment:

ID Search Hits

#1 MeSH descriptor: [Embryo Transfer] explode all trees 1529

#2 (Embryo Transfers):ti,ab,kw OR (Transfer, Embryo):ti,ab,kw OR (Transfers, Embryo):ti,ab,kw OR (Tubal Embryo Transfer):ti,ab,kw OR (Tubal Embryo Stage Transfer):ti,ab,kw OR (Blastocyst Transfer):ti,ab,kw 5900

#3 MeSH descriptor: [Reproductive Techniques, Assisted] explode all trees 4561

#4 (Assisted Reproductive Technique):ti,ab,kw OR (Reproductive Technique, Assisted):ti,ab,kw OR (Technique, Assisted Reproductive):ti,ab,kw OR (Techniques, Assisted Reproductive):ti,ab,kw OR (Assisted Reproductive Technics):ti,ab,kw OR (Assisted Reproductive Technic):ti,ab,kw OR (Reproductive Technic, Assisted):ti,ab,kw OR (Reproductive Technics, Assisted):ti,ab,kw OR (Technic, Assisted Reproductive):ti,ab,kw OR (Technics, Assisted Reproductive):ti,ab,kw OR (Assisted Reproductive Techniques):ti,ab,kw OR (Reproductive Technology, Assisted):ti,ab,kw OR (Assisted Reproductive Technologies):ti,ab,kw OR (Assisted Reproductive Technology):ti,ab,kw OR (Reproductive Technologies, Assisted):ti,ab,kw OR (Technologies, Assisted Reproductive):ti,ab,kw OR (Technology, Assisted Reproductive):ti,ab,kw 1583

#5 MeSH descriptor: [Fertilization in Vitro] explode all trees 2910

#6 (In Vitro Fertilization):ti,ab,kw OR (In Vitro Fertilizations):ti,ab,kw OR (Test-Tube Fertilization):ti,ab,kw OR (Fertilizations, Test-Tube):ti,ab,kw OR (Fertilization, Test-Tube):ti,ab,kw OR (Test Tube Fertilization):ti,ab,kw OR (Test-Tube Fertilizations):ti,ab,kw OR (Fertilizations in Vitro):ti,ab,kw OR (Test-Tube Babies):ti,ab,kw OR (Babies, Test-Tube):ti,ab,kw OR (Baby, Test-Tube):ti,ab,kw OR (Test Tube Babies):ti,ab,kw OR (Test-Tube Baby):ti,ab,kw 5789

#7 MeSH descriptor: [Oocytes] explode all trees 633

#8 (Oocyte):ti,ab,kw OR (Ovocytes):ti,ab,kw OR (Ovocyte):ti,ab,kw 4842

#9 #1 OR #2 5900

#10 #3 OR #4 5638

#11 #5 OR #6 6036

#12 #7 OR #8 4985

#13 #9 OR #10 OR #11 OR #12 12065

#14 MeSH descriptor: [Acupuncture] explode all trees 216

#15 (Pharmacopuncture):ti,ab,kw 120

#16 MeSH descriptor: [Acupuncture Therapy] explode all trees 7123

#17 (Acupuncture Treatment):ti,ab,kw OR (Acupuncture Treatments):ti,ab,kw OR (Treatment, Acupuncture):ti,ab,kw OR (Therapy, Acupuncture):ti,ab,kw OR (Pharmacoacupuncture Treatment):ti,ab,kw OR (Treatment, Pharmacoacupuncture):ti,ab,kw OR (Pharmacoacupuncture Therapy):ti,ab,kw OR (Therapy, Pharmacoacupuncture):ti,ab,kw OR (Acupotomy):ti,ab,kw OR (Acupotomies):ti,ab,kw 17357

#18 MeSH descriptor: [Auriculotherapy] explode all trees 328

#19 (Auriculotherapies):ti,ab,kw 0

#20 MeSH descriptor: [Moxibustion] explode all trees 688

#21 (Moxabustion):ti,ab,kw 0

#22 (Oriental Traditional Medicine):ti,ab,kw OR (Oriental Medicine, Traditional):ti,ab,kw OR (Medicine, Traditional Oriental):ti,ab,kw OR (Traditional Oriental Medicine):ti,ab,kw OR (Traditional Oriental Medicines):ti,ab,kw OR (Traditional Medicine, Oriental):ti,ab,kw OR (Medicine, Oriental Traditional):ti,ab,kw OR (Medicine, Traditional, East Asia):ti,ab,kw OR (Traditional Medicine, East Asia):ti,ab,kw OR (Traditional Far Eastern Medicine):ti,ab,kw OR (East Asian Traditional Medicine):ti,ab,kw OR (Traditional East Asian Medicine):ti,ab,kw OR (East Asian Medicine):ti,ab,kw OR (East Asian Medicines):ti,ab,kw OR (Medicine, East Asian):ti,ab,kw OR (Medicine, Oriental):ti,ab,kw OR (Oriental Medicine):ti,ab,kw OR (Medicine, East Asia):ti,ab,kw OR (Asia Medicines, East):ti,ab,kw OR (East Asia Medicine):ti,ab,kw OR (East Asia Medicines):ti,ab,kw OR (Medicines, East Asia):ti,ab,kw OR (Medicine, Far East):ti,ab,kw OR (East Medicine, Far):ti,ab,kw OR (East Medicines, Far):ti,ab,kw OR (Far East Medicine):ti,ab,kw OR (Far East Medicines):ti,ab,kw OR (Medicines, Far East):ti,ab,kw 347

#23 MeSH descriptor: [Medicine, Chinese Traditional] explode all trees 1806

#24 (Zhong Yi Xue):ti,ab,kw OR (Chung I Hsueh):ti,ab,kw OR (Hsueh, Chung I):ti,ab,kw OR (Traditional Medicine, Chinese):ti,ab,kw OR (Chinese Traditional Medicine):ti,ab,kw OR (Traditional Chinese Medicine):ti,ab,kw OR (Chinese Medicine, Traditional):ti,ab,kw OR (Traditional Tongue Diagnosis):ti,ab,kw OR (Tongue Diagnoses, Traditional):ti,ab,kw OR (Tongue Diagnosis, Traditional):ti,ab,kw OR (Traditional Tongue Diagnoses):ti,ab,kw OR (Traditional Tongue Assessment):ti,ab,kw OR (Tongue Assessment, Traditional):ti,ab,kw OR (Traditional Tongue Assessments):ti,ab,kw 12209

#25 MeSH descriptor: [Electroacupuncture] explode all trees 1172

#26 #14 OR #15 326

#27 #16 OR #17 18050

#28 #18 OR #19 328

#29 #20 OR #21 688

#30 #22 OR #23 OR #24 12737

#31 #25 OR #26 OR #27 OR #28 OR #29 OR #30 28767

#32 #13 AND #31 297
