# Supplementary material for: Acupuncture as an adjunctive therapy on embryo transfer day: a systematic review and meta-analysis of clinical pregnancy and live birth outcomes
Source: Front Reprod Health. 2025 Sep 23;7:1673144. doi: 10.3389/frph.2025.1673144 (PMC12500596; doi:10.3389/frph.2025.1673144)
Supplement: Supplementary file 3 [file Datasheet3.docx]

[Embase](http://www.embase.j.sjuku.top)

# Embase session results (18 May 2025)

| No. | Query | Results |
| --- | --- | --- |
| #34 | (('embryo transfer'/exp OR ('embryo transfers':ab,ti OR 'transfer, embryo':ab,ti OR 'transfers, embryo':ab,ti OR 'tubal embryo transfer':ab,ti OR 'tubal embryo stage transfer':ab,ti OR 'blastocyst transfer':ab,ti)) OR ('infertility therapy'/exp OR ('assisted reproductive technique':ab,ti OR 'reproductive technique, assisted':ab,ti OR 'technique, assisted reproductive':ab,ti OR 'techniques, assisted reproductive':ab,ti OR 'assisted reproductive technics':ab,ti OR 'assisted reproductive technic':ab,ti OR 'reproductive technic, assisted':ab,ti OR 'reproductive technics, assisted':ab,ti OR 'technic, assisted reproductive':ab,ti OR 'technics, assisted reproductive':ab,ti OR 'assisted reproductive techniques':ab,ti OR 'reproductive technology, assisted':ab,ti OR 'assisted reproductive technologies':ab,ti OR 'assisted reproductive technology':ab,ti OR 'reproductive technologies, assisted':ab,ti OR 'technologies, assisted reproductive':ab,ti OR 'technology, assisted reproductive':ab,ti OR 'reproductive techniques, assisted':ab,ti)) OR ('in vitro fertilization'/exp OR ('fertilization in vitro':ab,ti OR 'in vitro fertilizations':ab,ti OR 'test-tube fertilization':ab,ti OR 'fertilizations, test-tube':ab,ti OR 'fertilization, test-tube':ab,ti OR 'test tube fertilization':ab,ti OR 'test-tube fertilizations':ab,ti OR 'fertilizations in vitro':ab,ti OR 'test-tube babies':ab,ti OR 'babies, test-tube':ab,ti OR 'baby, test-tube':ab,ti OR 'test tube babies':ab,ti OR 'test-tube baby':ab,ti)) OR ('oocyte'/exp OR ('oocytes':ab,ti OR 'ovocytes':ab,ti OR 'ovocyte':ab,ti))) AND ('electroacupuncture'/exp OR ('acupuncture'/exp OR ('pharmacopuncture':ab,ti OR 'acupuncture treatment':ab,ti OR 'acupuncture treatments':ab,ti OR 'treatment, acupuncture':ab,ti OR 'therapy, acupuncture':ab,ti OR 'pharmacoacupuncture treatment':ab,ti OR 'treatment, pharmacoacupuncture':ab,ti OR 'pharmacoacupuncture therapy':ab,ti OR 'therapy, pharmacoacupuncture':ab,ti OR 'acupotomy':ab,ti OR 'acupotomies':ab,ti OR 'acupuncture therapy':ab,ti)) OR ('auricular acupuncture'/exp OR ('auriculotherapies':ab,ti OR 'auriculotherapy':ab,ti)) OR ('moxibustion'/exp OR 'moxabustion':ab,ti) OR ('oriental medicine'/exp OR ('oriental traditional medicine':ab,ti OR 'oriental medicine, traditional':ab,ti OR 'medicine, traditional oriental':ab,ti OR 'traditional oriental medicine':ab,ti OR 'traditional oriental medicines':ab,ti OR 'traditional medicine, oriental':ab,ti OR 'medicine, oriental traditional':ab,ti OR 'medicine, traditional, east asia':ab,ti OR 'traditional medicine, east asia':ab,ti OR 'traditional far eastern medicine':ab,ti OR 'east asian traditional medicine':ab,ti OR 'traditional east asian medicine':ab,ti OR 'east asian medicine':ab,ti OR 'east asian medicines':ab,ti OR 'medicine, east asian':ab,ti OR 'medicine, oriental':ab,ti OR 'medicine,oriental traditional':ab,ti OR 'medicine, east asia':ab,ti OR 'asia medicines, east':ab,ti OR 'east asia medicine':ab,ti OR 'east asia medicines':ab,ti OR 'medicines, east asia':ab,ti OR 'medicine, far east':ab,ti OR 'east medicine, far':ab,ti OR 'east medicines, far':ab,ti OR 'far east medicine':ab,ti OR 'far east medicines':ab,ti OR 'medicines, far east':ab,ti)) OR ('chinese medicine'/exp OR ('zhong yi xue':ab,ti OR 'chung i hsueh':ab,ti OR 'hsueh, chung i':ab,ti OR 'traditional medicine, chinese':ab,ti OR 'chinese traditional medicine':ab,ti OR 'traditional chinese medicine':ab,ti OR 'chinese medicine, traditional':ab,ti OR 'traditional tongue diagnosis':ab,ti OR 'tongue diagnoses, traditional':ab,ti OR 'tongue diagnosis, traditional':ab,ti OR 'traditional tongue diagnoses':ab,ti OR 'traditional tongue assessment':ab,ti OR 'tongue assessment, traditional':ab,ti OR 'traditional tongue assessments':ab,ti OR 'medicine, chinese traditional':ab,ti))) AND ('randomized controlled trial'/exp OR ('randomized':ab,ti OR 'placebo':ab,ti)) | 389 |
| #33 | 'randomized controlled trial'/exp OR ('randomized':ab,ti OR 'placebo':ab,ti) | 1652180 |
| #32 | 'randomized':ab,ti OR 'placebo':ab,ti | 1347489 |
| #31 | 'randomized controlled trial'/exp | 960690 |
| #30 | 'electroacupuncture'/exp OR ('acupuncture'/exp OR ('pharmacopuncture':ab,ti OR 'acupuncture treatment':ab,ti OR 'acupuncture treatments':ab,ti OR 'treatment, acupuncture':ab,ti OR 'therapy, acupuncture':ab,ti OR 'pharmacoacupuncture treatment':ab,ti OR 'treatment, pharmacoacupuncture':ab,ti OR 'pharmacoacupuncture therapy':ab,ti OR 'therapy, pharmacoacupuncture':ab,ti OR 'acupotomy':ab,ti OR 'acupotomies':ab,ti OR 'acupuncture therapy':ab,ti)) OR ('auricular acupuncture'/exp OR ('auriculotherapies':ab,ti OR 'auriculotherapy':ab,ti)) OR ('moxibustion'/exp OR 'moxabustion':ab,ti) OR ('oriental medicine'/exp OR ('oriental traditional medicine':ab,ti OR 'oriental medicine, traditional':ab,ti OR 'medicine, traditional oriental':ab,ti OR 'traditional oriental medicine':ab,ti OR 'traditional oriental medicines':ab,ti OR 'traditional medicine, oriental':ab,ti OR 'medicine, oriental traditional':ab,ti OR 'medicine, traditional, east asia':ab,ti OR 'traditional medicine, east asia':ab,ti OR 'traditional far eastern medicine':ab,ti OR 'east asian traditional medicine':ab,ti OR 'traditional east asian medicine':ab,ti OR 'east asian medicine':ab,ti OR 'east asian medicines':ab,ti OR 'medicine, east asian':ab,ti OR 'medicine, oriental':ab,ti OR 'medicine,oriental traditional':ab,ti OR 'medicine, east asia':ab,ti OR 'asia medicines, east':ab,ti OR 'east asia medicine':ab,ti OR 'east asia medicines':ab,ti OR 'medicines, east asia':ab,ti OR 'medicine, far east':ab,ti OR 'east medicine, far':ab,ti OR 'east medicines, far':ab,ti OR 'far east medicine':ab,ti OR 'far east medicines':ab,ti OR 'medicines, far east':ab,ti)) OR ('chinese medicine'/exp OR ('zhong yi xue':ab,ti OR 'chung i hsueh':ab,ti OR 'hsueh, chung i':ab,ti OR 'traditional medicine, chinese':ab,ti OR 'chinese traditional medicine':ab,ti OR 'traditional chinese medicine':ab,ti OR 'chinese medicine, traditional':ab,ti OR 'traditional tongue diagnosis':ab,ti OR 'tongue diagnoses, traditional':ab,ti OR 'tongue diagnosis, traditional':ab,ti OR 'traditional tongue diagnoses':ab,ti OR 'traditional tongue assessment':ab,ti OR 'tongue assessment, traditional':ab,ti OR 'traditional tongue assessments':ab,ti OR 'medicine, chinese traditional':ab,ti)) | 166901 |
| #29 | 'chinese medicine'/exp OR ('zhong yi xue':ab,ti OR 'chung i hsueh':ab,ti OR 'hsueh, chung i':ab,ti OR 'traditional medicine, chinese':ab,ti OR 'chinese traditional medicine':ab,ti OR 'traditional chinese medicine':ab,ti OR 'chinese medicine, traditional':ab,ti OR 'traditional tongue diagnosis':ab,ti OR 'tongue diagnoses, traditional':ab,ti OR 'tongue diagnosis, traditional':ab,ti OR 'traditional tongue diagnoses':ab,ti OR 'traditional tongue assessment':ab,ti OR 'tongue assessment, traditional':ab,ti OR 'traditional tongue assessments':ab,ti OR 'medicine, chinese traditional':ab,ti) | 107735 |
| #28 | 'oriental medicine'/exp OR ('oriental traditional medicine':ab,ti OR 'oriental medicine, traditional':ab,ti OR 'medicine, traditional oriental':ab,ti OR 'traditional oriental medicine':ab,ti OR 'traditional oriental medicines':ab,ti OR 'traditional medicine, oriental':ab,ti OR 'medicine, oriental traditional':ab,ti OR 'medicine, traditional, east asia':ab,ti OR 'traditional medicine, east asia':ab,ti OR 'traditional far eastern medicine':ab,ti OR 'east asian traditional medicine':ab,ti OR 'traditional east asian medicine':ab,ti OR 'east asian medicine':ab,ti OR 'east asian medicines':ab,ti OR 'medicine, east asian':ab,ti OR 'medicine, oriental':ab,ti OR 'medicine,oriental traditional':ab,ti OR 'medicine, east asia':ab,ti OR 'asia medicines, east':ab,ti OR 'east asia medicine':ab,ti OR 'east asia medicines':ab,ti OR 'medicines, east asia':ab,ti OR 'medicine, far east':ab,ti OR 'east medicine, far':ab,ti OR 'east medicines, far':ab,ti OR 'far east medicine':ab,ti OR 'far east medicines':ab,ti OR 'medicines, far east':ab,ti) | 3816 |
| #27 | 'moxibustion'/exp OR 'moxabustion':ab,ti | 5440 |
| #26 | 'auricular acupuncture'/exp OR ('auriculotherapies':ab,ti OR 'auriculotherapy':ab,ti) | 1392 |
| #25 | 'acupuncture'/exp OR ('pharmacopuncture':ab,ti OR 'acupuncture treatment':ab,ti OR 'acupuncture treatments':ab,ti OR 'treatment, acupuncture':ab,ti OR 'therapy, acupuncture':ab,ti OR 'pharmacoacupuncture treatment':ab,ti OR 'treatment, pharmacoacupuncture':ab,ti OR 'pharmacoacupuncture therapy':ab,ti OR 'therapy, pharmacoacupuncture':ab,ti OR 'acupotomy':ab,ti OR 'acupotomies':ab,ti OR 'acupuncture therapy':ab,ti) | 64959 |
| #24 | 'zhong yi xue':ab,ti OR 'chung i hsueh':ab,ti OR 'hsueh, chung i':ab,ti OR 'traditional medicine, chinese':ab,ti OR 'chinese traditional medicine':ab,ti OR 'traditional chinese medicine':ab,ti OR 'chinese medicine, traditional':ab,ti OR 'traditional tongue diagnosis':ab,ti OR 'tongue diagnoses, traditional':ab,ti OR 'tongue diagnosis, traditional':ab,ti OR 'traditional tongue diagnoses':ab,ti OR 'traditional tongue assessment':ab,ti OR 'tongue assessment, traditional':ab,ti OR 'traditional tongue assessments':ab,ti OR 'medicine, chinese traditional':ab,ti | 52245 |
| #23 | 'chinese medicine'/exp | 89979 |
| #22 | 'oriental traditional medicine':ab,ti OR 'oriental medicine, traditional':ab,ti OR 'medicine, traditional oriental':ab,ti OR 'traditional oriental medicine':ab,ti OR 'traditional oriental medicines':ab,ti OR 'traditional medicine, oriental':ab,ti OR 'medicine, oriental traditional':ab,ti OR 'medicine, traditional, east asia':ab,ti OR 'traditional medicine, east asia':ab,ti OR 'traditional far eastern medicine':ab,ti OR 'east asian traditional medicine':ab,ti OR 'traditional east asian medicine':ab,ti OR 'east asian medicine':ab,ti OR 'east asian medicines':ab,ti OR 'medicine, east asian':ab,ti OR 'medicine, oriental':ab,ti OR 'medicine,oriental traditional':ab,ti OR 'medicine, east asia':ab,ti OR 'asia medicines, east':ab,ti OR 'east asia medicine':ab,ti OR 'east asia medicines':ab,ti OR 'medicines, east asia':ab,ti OR 'medicine, far east':ab,ti OR 'east medicine, far':ab,ti OR 'east medicines, far':ab,ti OR 'far east medicine':ab,ti OR 'far east medicines':ab,ti OR 'medicines, far east':ab,ti | 791 |
| #21 | 'oriental medicine'/exp | 3188 |
| #20 | 'moxabustion':ab,ti | 2 |
| #19 | 'moxibustion'/exp | 5438 |
| #18 | 'electroacupuncture'/exp | 11032 |
| #17 | 'auriculotherapies':ab,ti OR 'auriculotherapy':ab,ti | 402 |
| #16 | 'auricular acupuncture'/exp | 1173 |
| #15 | 'pharmacopuncture':ab,ti OR 'acupuncture treatment':ab,ti OR 'acupuncture treatments':ab,ti OR 'treatment, acupuncture':ab,ti OR 'therapy, acupuncture':ab,ti OR 'pharmacoacupuncture treatment':ab,ti OR 'treatment, pharmacoacupuncture':ab,ti OR 'pharmacoacupuncture therapy':ab,ti OR 'therapy, pharmacoacupuncture':ab,ti OR 'acupotomy':ab,ti OR 'acupotomies':ab,ti OR 'acupuncture therapy':ab,ti | 8885 |
| #14 | 'acupuncture'/exp | 64539 |
| #13 | ('embryo transfer'/exp OR ('embryo transfers':ab,ti OR 'transfer, embryo':ab,ti OR 'transfers, embryo':ab,ti OR 'tubal embryo transfer':ab,ti OR 'tubal embryo stage transfer':ab,ti OR 'blastocyst transfer':ab,ti)) OR ('infertility therapy'/exp OR ('assisted reproductive technique':ab,ti OR 'reproductive technique, assisted':ab,ti OR 'technique, assisted reproductive':ab,ti OR 'techniques, assisted reproductive':ab,ti OR 'assisted reproductive technics':ab,ti OR 'assisted reproductive technic':ab,ti OR 'reproductive technic, assisted':ab,ti OR 'reproductive technics, assisted':ab,ti OR 'technic, assisted reproductive':ab,ti OR 'technics, assisted reproductive':ab,ti OR 'assisted reproductive techniques':ab,ti OR 'reproductive technology, assisted':ab,ti OR 'assisted reproductive technologies':ab,ti OR 'assisted reproductive technology':ab,ti OR 'reproductive technologies, assisted':ab,ti OR 'technologies, assisted reproductive':ab,ti OR 'technology, assisted reproductive':ab,ti OR 'reproductive techniques, assisted':ab,ti)) OR ('in vitro fertilization'/exp OR ('fertilization in vitro':ab,ti OR 'in vitro fertilizations':ab,ti OR 'test-tube fertilization':ab,ti OR 'fertilizations, test-tube':ab,ti OR 'fertilization, test-tube':ab,ti OR 'test tube fertilization':ab,ti OR 'test-tube fertilizations':ab,ti OR 'fertilizations in vitro':ab,ti OR 'test-tube babies':ab,ti OR 'babies, test-tube':ab,ti OR 'baby, test-tube':ab,ti OR 'test tube babies':ab,ti OR 'test-tube baby':ab,ti)) OR ('oocyte'/exp OR ('oocytes':ab,ti OR 'ovocytes':ab,ti OR 'ovocyte':ab,ti)) | 265347 |
| #12 | 'oocyte'/exp OR ('oocytes':ab,ti OR 'ovocytes':ab,ti OR 'ovocyte':ab,ti) | 125632 |
| #11 | 'in vitro fertilization'/exp OR ('fertilization in vitro':ab,ti OR 'in vitro fertilizations':ab,ti OR 'test-tube fertilization':ab,ti OR 'fertilizations, test-tube':ab,ti OR 'fertilization, test-tube':ab,ti OR 'test tube fertilization':ab,ti OR 'test-tube fertilizations':ab,ti OR 'fertilizations in vitro':ab,ti OR 'test-tube babies':ab,ti OR 'babies, test-tube':ab,ti OR 'baby, test-tube':ab,ti OR 'test tube babies':ab,ti OR 'test-tube baby':ab,ti) | 127539 |
| #10 | 'infertility therapy'/exp OR ('assisted reproductive technique':ab,ti OR 'reproductive technique, assisted':ab,ti OR 'technique, assisted reproductive':ab,ti OR 'techniques, assisted reproductive':ab,ti OR 'assisted reproductive technics':ab,ti OR 'assisted reproductive technic':ab,ti OR 'reproductive technic, assisted':ab,ti OR 'reproductive technics, assisted':ab,ti OR 'technic, assisted reproductive':ab,ti OR 'technics, assisted reproductive':ab,ti OR 'assisted reproductive techniques':ab,ti OR 'reproductive technology, assisted':ab,ti OR 'assisted reproductive technologies':ab,ti OR 'assisted reproductive technology':ab,ti OR 'reproductive technologies, assisted':ab,ti OR 'technologies, assisted reproductive':ab,ti OR 'technology, assisted reproductive':ab,ti OR 'reproductive techniques, assisted':ab,ti) | 184142 |
| #9 | 'embryo transfer'/exp OR ('embryo transfers':ab,ti OR 'transfer, embryo':ab,ti OR 'transfers, embryo':ab,ti OR 'tubal embryo transfer':ab,ti OR 'tubal embryo stage transfer':ab,ti OR 'blastocyst transfer':ab,ti) | 46367 |
| #8 | 'oocytes':ab,ti OR 'ovocytes':ab,ti OR 'ovocyte':ab,ti | 84807 |
| #7 | 'oocyte'/exp | 96748 |
| #6 | 'fertilization in vitro':ab,ti OR 'in vitro fertilizations':ab,ti OR 'test-tube fertilization':ab,ti OR 'fertilizations, test-tube':ab,ti OR 'fertilization, test-tube':ab,ti OR 'test tube fertilization':ab,ti OR 'test-tube fertilizations':ab,ti OR 'fertilizations in vitro':ab,ti OR 'test-tube babies':ab,ti OR 'babies, test-tube':ab,ti OR 'baby, test-tube':ab,ti OR 'test tube babies':ab,ti OR 'test-tube baby':ab,ti | 1126 |
| #5 | 'in vitro fertilization'/exp | 127306 |
| #4 | 'assisted reproductive technique':ab,ti OR 'reproductive technique, assisted':ab,ti OR 'technique, assisted reproductive':ab,ti OR 'techniques, assisted reproductive':ab,ti OR 'assisted reproductive technics':ab,ti OR 'assisted reproductive technic':ab,ti OR 'reproductive technic, assisted':ab,ti OR 'reproductive technics, assisted':ab,ti OR 'technic, assisted reproductive':ab,ti OR 'technics, assisted reproductive':ab,ti OR 'assisted reproductive techniques':ab,ti OR 'reproductive technology, assisted':ab,ti OR 'assisted reproductive technologies':ab,ti OR 'assisted reproductive technology':ab,ti OR 'reproductive technologies, assisted':ab,ti OR 'technologies, assisted reproductive':ab,ti OR 'technology, assisted reproductive':ab,ti OR 'reproductive techniques, assisted':ab,ti | 21125 |
| #3 | 'infertility therapy'/exp | 180611 |
| #2 | 'embryo transfers':ab,ti OR 'transfer, embryo':ab,ti OR 'transfers, embryo':ab,ti OR 'tubal embryo transfer':ab,ti OR 'tubal embryo stage transfer':ab,ti OR 'blastocyst transfer':ab,ti | 8137 |
| #1 | 'embryo transfer'/exp | 45296 |

Copyright © 2025 Elsevier Limited except certain content provided by third parties.

Embase is a trade mark of Elsevier Life Sciences IP Limited.
